# Recent Advances in Artificial Sensory Neurons: Biological Fundamentals, Devices, Applications, and Challenges

**DOI:** 10.1007/s40820-024-01550-x

**Published:** 2024-11-13

**Authors:** Shuai Zhong, Lirou Su, Mingkun Xu, Desmond Loke, Bin Yu, Yishu Zhang, Rong Zhao

**Affiliations:** 1 Guangdong Institute of Intelligence Science and Technology, Hengqin, Zhuhai 519031 People’s Republic of China; 2 College of Integrated Circuits, Zhejiang University, Hangzhou, 3112000 People’s Republic of China; 3 ZJU-Hangzhou Global Scientific and Technological Innovation Center, Hangzhou, 310027 People’s Republic of China; 4 Department of Science, Mathematics and Technology, Singapore University of Technology and Design, Singapore, 487372 Singapore; 5 Department of Precision Instruments, Tsinghua University, Beijing, 100084 People’s Republic of China; 6 Center for Brain-Inspired Computing Research, Tsinghua University, Beijing, 100084 People’s Republic of China; 7 IDG/McGovern Institute for Brain Research, Tsinghua University, Beijing, 100084 People’s Republic of China

**Keywords:** Artificial intelligence, Emerging devices, Artificial sensory neurons, Spiking neural networks, Neuromorphic sensing

## Abstract

Spike-based neural networks, which use spikes or action potentials to represent information, have gained a lot of attention because of their high energy efficiency and low power consumption. To fully leverage its advantages, converting the external analog signals to spikes is an essential prerequisite. Conventional approaches including analog-to-digital converters or ring oscillators, and sensors suffer from high power and area costs. Recent efforts are devoted to constructing artificial sensory neurons based on emerging devices inspired by the biological sensory system. They can simultaneously perform sensing and spike conversion, overcoming the deficiencies of traditional sensory systems. This review summarizes and benchmarks the recent progress of artificial sensory neurons. It starts with the presentation of various mechanisms of biological signal transduction, followed by the systematic introduction of the emerging devices employed for artificial sensory neurons. Furthermore, the implementations with different perceptual capabilities are briefly outlined and the key metrics and potential applications are also provided. Finally, we highlight the challenges and perspectives for the future development of artificial sensory neurons.

## Highlights

- Biological fundamentals and recent progress of artificial sensory neurons are systematically reviewed.
- Basic device, performance metrics, and potential applications of artificial sensory neurons are summarized.
- Challenges for the future development of artificial sensory neurons are discussed.

## Graphical abstract

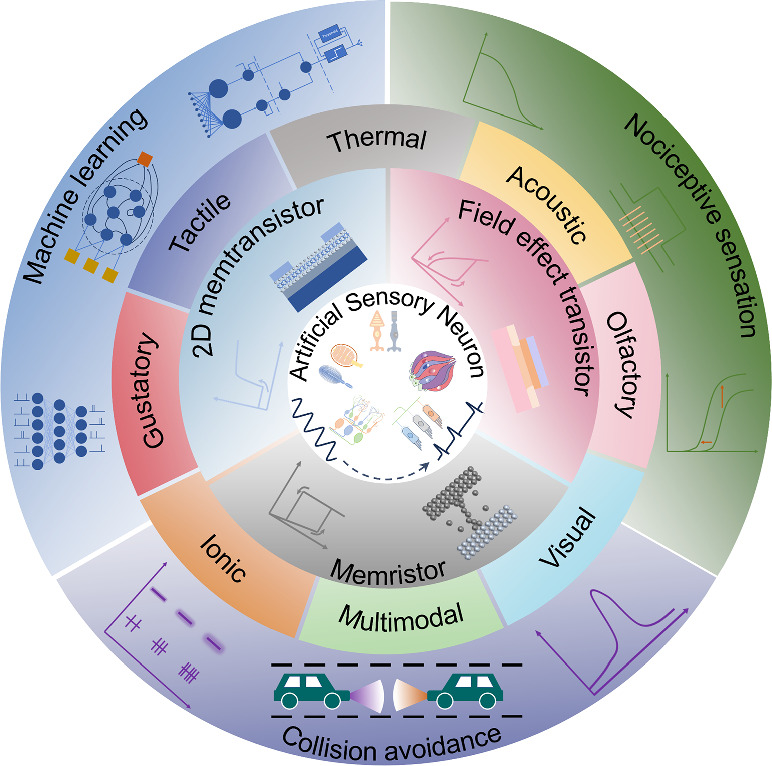

## Introduction

Artificial intelligence (AI) has significantly propelled the advancement of human society, including fields such as healthcare, transportation, manufacturing, and entertainment [1–5]. The recent release of ChatGPT shows remarkable performance in interactive dialogue generation, confirming the fact that the era of AI is approaching. The realization of AI relies heavily on computing hardware, which is widely utilized with conventional von Neumann architecture [6, 7]. However, there is a well-known bottleneck in von Neumann architecture due to the separation of computing and memory units [8, 9]. Frequent data transmission between these units is required, leading to decreased computing speed and increased energy consumption. For applications such as edge computing and the Internet of Things (IoT), where energy efficiency is paramount, there is a pressing demand for new computing architectures [10–12].

Neuromorphic computing, inspired by the structure and function of biological neural networks (BNNs), utilizes artificial neurons and synapses to process information [13–17]. It intends to surmount the von Neumann bottleneck, facilitating efficient and parallel data processing. Such architecture is particularly well-suited for tasks involving pattern recognition, sensory processing, and real-time data analysis. Spiking neural networks (SNNs), regarded as the third generation of artificial neural networks (ANNs), have garnered significant attention due to their high energy efficiency and spatiotemporal processing capabilities [18, 19]. In contrast to traditional ANNs, which employ continuous values to represent neuron activations, SNNs use discrete spikes or pulses to convey information, analogous to the BNNs. Specifically, in an SNN, neurons integrate incoming signals over time and when the integrated signals reach a certain threshold, the neuron generates a spike and transmits it to other neurons. Nowadays, various strategies have been proposed for the implementation of artificial neurons in SNNs [20]. Artificial neurons based on complementary metal–oxide–semiconductor (CMOS) technology have been applied in the TrueNorth chip (IBM) and the Loihi chip (Intel) owing to their excellent technology maturity [21, 22]. However, drawbacks such as poor scalability, limited neural dynamics, and high power consumption are inevitable. To address these issues, emerging memory devices are being extensively investigated for neuronal implementation, including memristors, insulator–metal transition (IMT) devices, phase change memories (PCM), ferroelectric field-effect transistors (FeFET), magnetic skyrmionics devices, and ovonic threshold switching (OTS) devices and so on [23–26]. Moreover, SNN hardware by co-integrating novel neuronal and synaptic devices has also been successfully demonstrated [27, 28].

Spike information in discrete representation is integral to the training and operation of SNNs. However, sensory data collected from the environment by various sensors, are typically analog in nature and thus cannot be directly fed into the SNN for processing. Consequently, there is a critical need for specialized devices capable of converting analog signals into spikes. One conventional method to address this challenge is the utilization of analog-to-digital converters (ADCs). ADCs operate by sampling the analog signal at regular intervals and subsequently quantizing each sample to produce a digital value. The attributes of noise immunity, ease of integration, and flexibility have contributed to their widespread adoption in modern electronic systems [29]. In addition to ADCs, ring oscillators have also been employed to translate analog signals into spike trains. A ring oscillator functions by employing a series of inverting amplifier stages connected in a loop, thereby creating a feedback system that sustains oscillation. As the signal propagates through each stage, it experiences a phase shift and gain, which are carefully designed to maintain a constant waveform. The number of stages and their arrangement determine the frequency and complexity of the output signal. The oscillator’s capacity to produce a stable, repetitive waveform renders it useful in diverse applications, including timing circuits and signal generation [30–33]. Nonetheless, the power consumption of both ADCs and ring oscillators remains a significant concern, particularly in scenarios requiring high resolution and high speed. Furthermore, their complex circuit designs result in physical sizes that may be unfavorable in compact designs. Such limitation is exacerbated when multiple converters or oscillators must be integrated or when the design area is a primary constraint. Therefore, the development of devices that can efficiently and compactly convert signals from the analog domain to the spike domain is of paramount importance.

The biological sensory system processes external stimuli, such as physical touch, light, sound, and chemicals, in parallel with ultra-low power consumption. When a stimulus is detected by receptors, an electrical spike is generated and transmitted to the central nervous system (CNS) for decoding [34]. In recent years, drawing inspiration from the operating mechanism of the biological sensory system, emerging neuromorphic devices-based artificial sensory neurons (ASNs) that can efficiently convert environmental information into electrical spikes have been widely explored, aiming to overcome the limitations of conventional CMOS-based counterparts. Emerging neuromorphic devices generally offer high scalability, low power consumption, and high speed, which can reduce the energy and hardware costs associated with sensory transduction [35, 36]. The implementations of ASNs are highly rely on artificial neuronal devices and various sensors. Currently, a number of reviews provide a comprehensive summary on artificial neuronal devices from perspective of materials, neural dynamics, and applications [24, 37–41]. For example, Lee et al. [24] highlighted the 2D materials-based artificial neuronal and synaptic devices for next-generation neuromorphic computing. Liu et al. [37] reviewed the emerging volatile switching materials with a focus on the neuronal dynamics for computational and sensing applications. Wang et al. [40] surveyed various artificial neuronal devices for brain-computer interfaces and neuroscience research. However, in these reviews, ASNs are ignored or briefly discussed as an aspect of neuronal applications. It should be pointed out that ASNs are generally realized based on artificial neuronal devices, they are quite different in terms of biological fundamentals, working mechanism, performance metrics, potential applications, and future roadmap. ASNs imitate the behavior of biological receptors instead of biological neurons and function as sensors to detect the external stimulus. Their spiking dynamics is heavily contingent on the matching between the artificial neuronal devices and sensors. Because of their sensing capabilities, the assessment of ASNs is not limited to the spiking frequency, uniformity, stability and power consumption that we care about the artificial neuronal devices, but also includes properties such as sensitivity, dynamic range, linearity, resolution, response time and so on that related to sensors. The ASNs are expected to play their roles in neuromorphic sensing, while artificial neuronal devices are mostly used for neuromorphic computing. In light of this, the development roadmap of ASNs should be envisioned by integrating the evolution of artificial neuronal devices and sensors. Consequently, different from previous review reports, we present a comprehensive review on recent progress in ASNs from biological principle, various implementations, characteristic evaluation to future development, aiming to offer guidelines for the advancement of neuromorphic sensing and computing. Noting that the term “artificial sensory neuron” is not determinately defined and used to represent devices such as sensors with neuronal devices, sensors with artificial synapses, or sensors that incorporate both artificial neuronal and synaptic devices [42–46]. To clarify, here, we use ASNs to denote devices or systems capable of sensing external stimuli and converting them into spikes according to the biological description of sensory neurons [47–50].

**Fig. 1:**
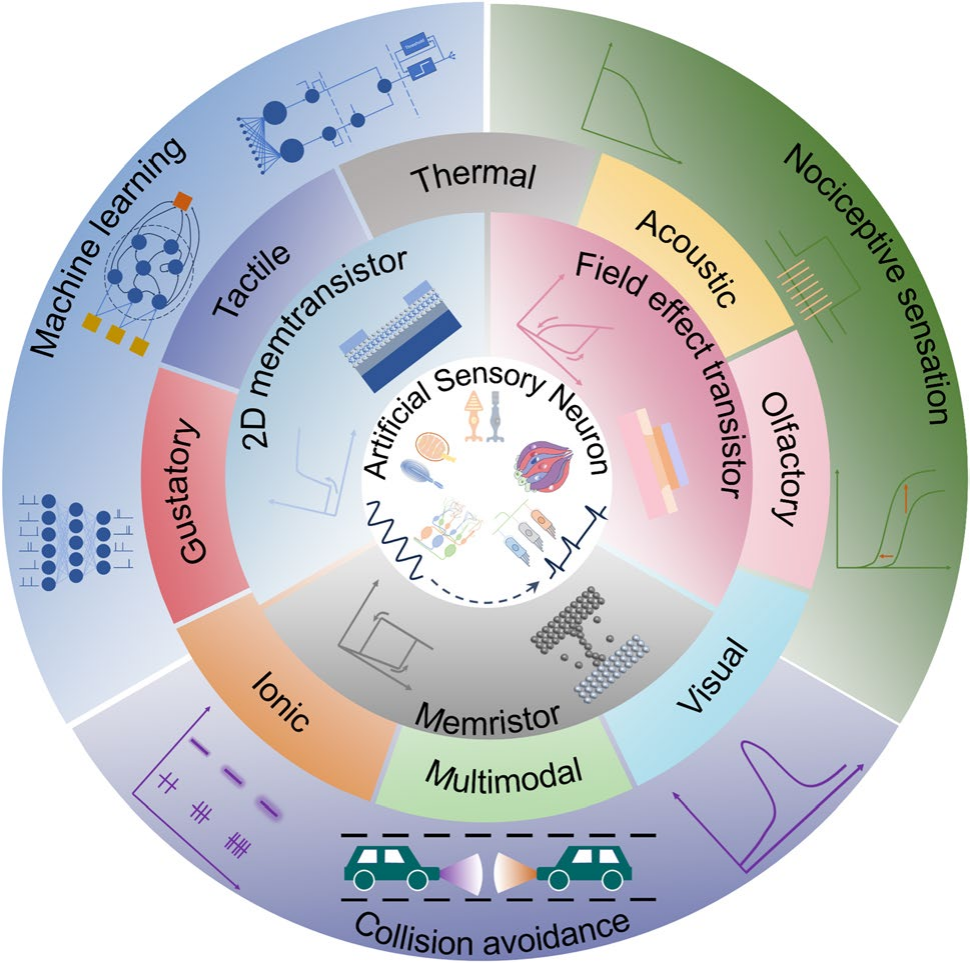
An overview of ASNs from biological fundamentals, devices to sensation implementation and applications

In this review, we survey the recent progress of ASNs for neuromorphic sensing and encoding (Fig. 1). We firstly introduce the working mechanism of different biological sensory receptors which may offer some insights for the development of ASNs. Then we summarize the devices normally used for ASNs, for example, memristor, 2D memtransistor, and field effect transistor. In addition, various categories of ASNs are presented including tactile, thermal, acoustic, olfactory, visual, gustatory, ionic, and multimodal sensation. Furthermore, the performance metrics such as energy consumption, linearity, sensitivity, and the potential applications of ASNs are provided. Finally, we discuss the challenges and perspectives of ASNs for practical application.

## Mechanism of Biological Signal Transduction

The encoding of external stimuli in humans is achieved via biological sensory receptors. They are specialized cells or structures within the sensory organ, which can detect specific types of incentives and convert them into electrical spike signals that can be interpreted by the CNS [51]. General types of sensory receptors, according to their functionality, encompass visual receptors, tactile receptors, thermal receptors, gustatory receptors, acoustic receptors, olfactory receptors, etc. These receptors help to perceive and adapt to changes in the environment with appropriate physiological responses. Deep understanding of working mechanism of biological receptors may offer some insights to the future development of ASNs.

### Visual Receptors

**Fig. 2:**
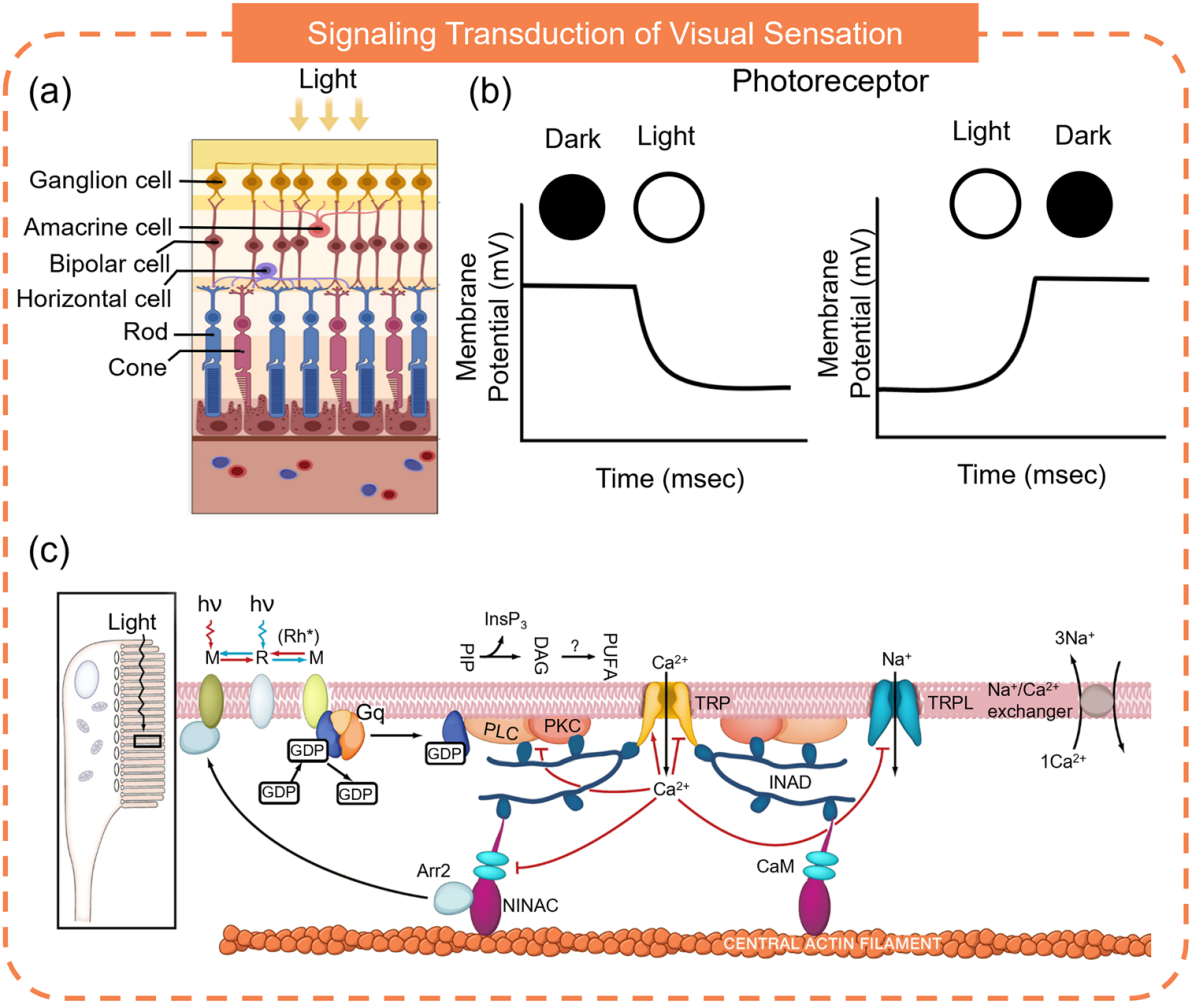
Biological visual system and molecular mechanism of visual encoding. **a** The structure of retina. Reproduced with permission from Ref. [52]. Copyright 2022, Elsevier. **b** Response of photoreceptor. **c** Process of phototransduction. Reproduced with permission from Ref. [54]. Copyright 2009, Elsevier

The visual sensory system is critically important, as approximately 80% of external information is received through the eyes. It enables the interpretation of environmental cues such as brightness, shape, and color, contributing to the decision-making. Additionally, it assists in navigating, avoiding potential dangers, and ensuring safety. The human eye comprises several structures, including the cornea, pupil, iris, lens, ciliary body, vitreous humor, choroid, sclera, and retina. Among them, the retina is responsible for light detection and transduction. The retina’s structure includes the pigmented epithelium layer, photoreceptor layer, bipolar cell layer, ganglion cell layer, nerve fiber layer, and supporting cells and blood vessels (Fig. 2a). There are two types of photoreceptors: rods and cones, which detect light and perform phototransduction [52, 53]. A move from dark to bright causes a hyperpolarization of photoreceptors and a move from bright to dark leads to a depolarization of photoreceptors (Fig. 2b). Specifically, light is absorbed by photopigments, causing a conformational change that transforms the photopigment from an inactive meta-rhodopsin state to an active meta-rhodopsin II state (Fig. 2c) [54]. This transformation activates the G-protein transducin, which is bound to the cytoplasmic surface of the photoreceptor cell membrane, leading to the release of guanosine-5’-diphosphate (GDP) and subsequent binding to guanosine-5’-triphosphate (GTP) [55]. This causes GTP to dissociate from the photopigment. The activated transducin and GTP interact with the phosphodiesterase (PDE) enzyme, catalyzing the hydrolysis of cyclic guanosine monophosphate (cGMP) into guanosine monophosphate (GMP). The cGMP is essential for maintaining the opening of cGMP-gated ion channels in the cell membrane. The decrease in cGMP concentration resulting from its breakdown leads to the closure of these ion channels. The closure prevents cation ions from entering the cell, resulting in hyperpolarization of the photoreceptors [56–58]. This hyperpolarization inhibits the release of neurotransmitters from photoreceptor cells to bipolar cells, thus reducing the electrical potential of bipolar cells. The process is reversed in the absence of light.

### Tactile/Thermal Receptors

**Fig. 3:**
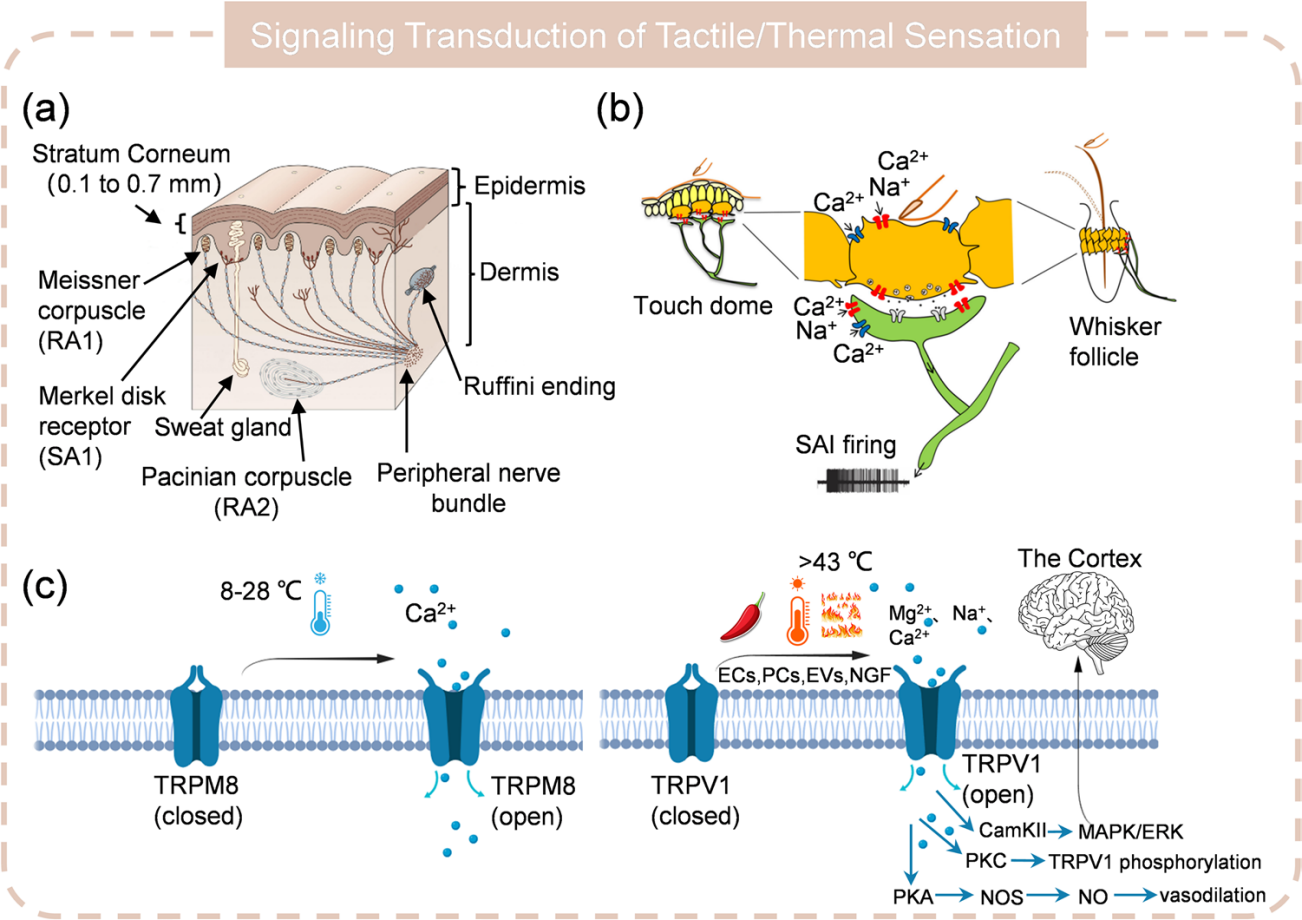
Signaling transduction of tactile/thermal sensation. **a** Cutaneous structure with mechanoreceptors. Reproduced with permission from Ref. [66]. Copyright 2022, Frontiers. **b** Tactile transduction in the Merkel cell-neurite complex. Reproduced with permission from Ref. [63]. Copyright 2014, Elsevier. **c** Thermal transduction at molecular level related to TRPM 8 and TRPV 1. Reproduced with permission from Ref. [73]. Multidisciplinary Digital Publishing Institute

The tactile sensory system is essential as it enables individuals to perceive and interpret physical contact, providing crucial information for object recognition, manipulation, and social interaction. Skin, with its complex and layered structure, is the primary organ to detect pressure, vibration, and temperature. Understanding how tactile information is perceived and encoded is vital. Recent progress has revealed that the mechanically sensitive cation channel PIEZO2 plays a significant role in mechanotransduction [59–62]. Within the cutaneous structure, various mechanoreceptors such as Merkel cells, detect mechanical stimuli and convert them into electrical spikes that are transmitted to the CNS for decoding (Fig. 3a) [63–66]. Mechanotransduction occurs as follows: when external pressure, vibration, or tension is applied to the skin, it deforms the cell membrane of mechanoreceptors, leading to the opening of mechanosensitive ion channels, such as PIEZO2 (Fig. 3b) [63]. The opening of PIEZO2 channels allows cation ions to enter the cells, causing depolarization. This depolarization activates voltage-gated calcium channels, which results in the release of neurotransmitters and the generation of action potentials [67]. However, the intricacies of mechanotransduction are still not fully understood because of the diversity of mechanoreceptors.

Thermal sensation is another aspect of tactile perception, allowing individuals to sense the temperature. This ability is crucial for adapting to environments and avoiding damage from temperature change [68]. Thermoreceptors are specialized receptors that detect temperature changes and convert them into electrical spikes [69]. It is well-reported that thermoreceptors containing the transient receptor potential melastatin 8 (TRPM8) ion channel are sensitive to cold temperatures (8 ~ 28 °C), while those with the transient receptor potential vanilloid 1 (TRPV1) channel contribute to the detection of warmth and heat (24 ~ 35 °C), as shown in Fig. 3c [70–73]. Both TRPM8 and TRPV1 are calcium-permeable channels, and when activated by cold or high temperatures, a conformational change in their structure occurs, opening the channels and allowing calcium ions to flow into the cell, which generates action potentials. The signals are then transmitted through dorsal root ganglia (DRG) to the cortex for processing. It should be noted that the molecular mechanisms underlying the activation of TRPM8 and TRPV1 by cold and heat are not fully understood, and active research in this area is ongoing.

### Gustatory Receptors

**Fig. 4:**
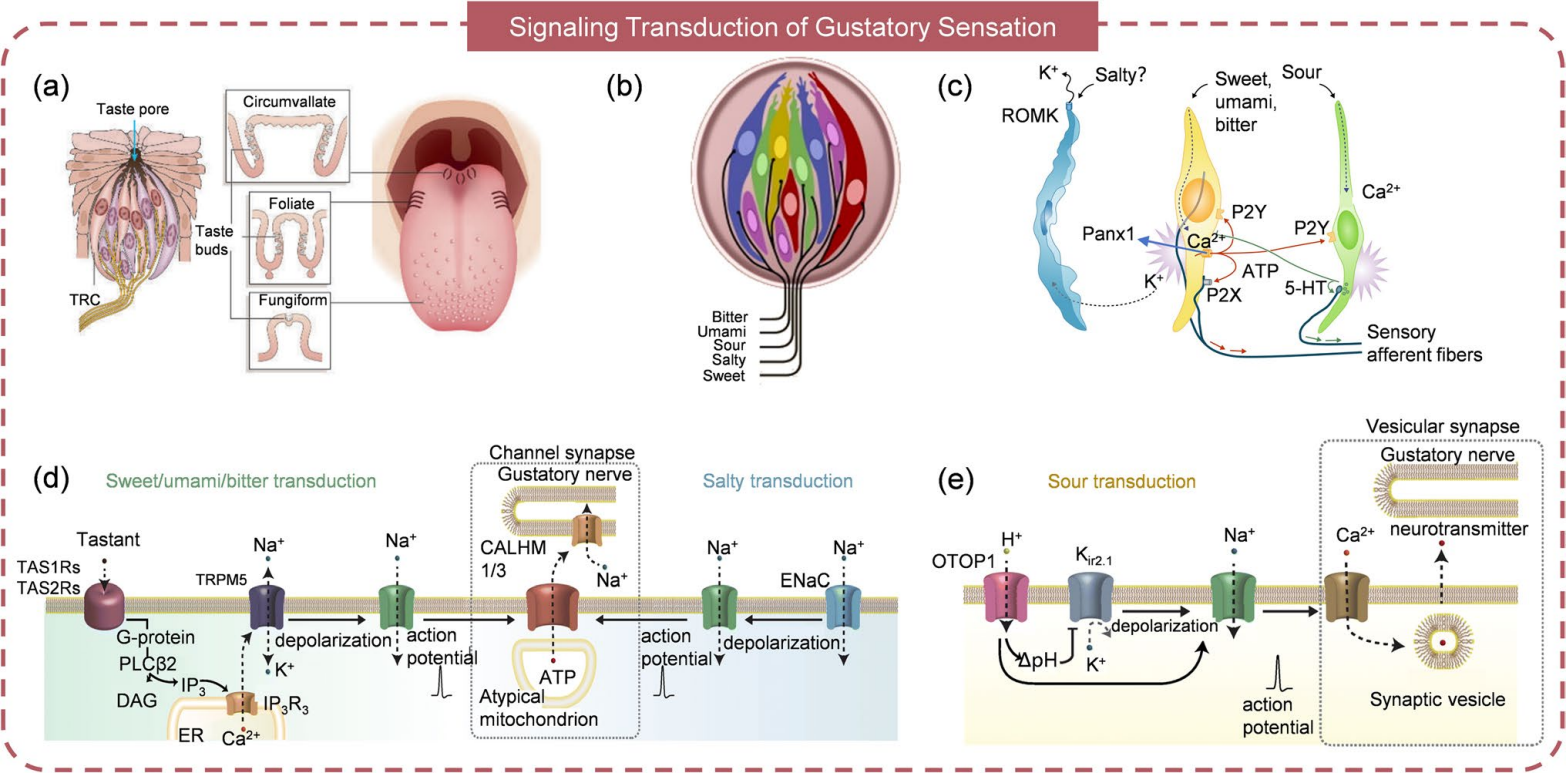
The mechanism of taste sensing and encoding. **a** Papillae distribution in tongue. Reproduced with permission from Ref. [75]. Copyright 2006, Springer Nature. **b** Taste receptor cells mediate taste sensation. Reproduced with permission from Ref. [77]. Copyright 2009, Elsevier. **c** Type I, II, and III receptor cells of taste. Reproduced with permission from Ref. [78]. Copyright 2010, Rockefeller University Press. **d** Process of sweet, umami, bitter and salty transduction. **e** Process of sour transduction. **d**–**e** Reproduced with permission from Ref. [79]. Copyright 2020, Springer-Verlag GmbH Germany

Taste is another sensory modality for humans, playing a pivotal role in food evaluation, the formation of dietary preferences, and the avoidance of potential toxins. Through tasting, we can accomplish recognition and differentiation of various flavors, including sweet, salty, sour, bitter, and umami sensations, which is instrumental in assessing the nutritional value, freshness, and quality of foods and beverages. Additionally, taste is closely linked to the pleasure or displeasure experienced during eating. The primary structure on the tongue for taste detection is the papillae [74, 75]. Fungiform papillae are small, club-shaped structures concentrated in the middle region of the tongue; foliate papillae are leaf-shaped and located on the sides at the back of the tongue; circumvallate papillae, surrounded by a circular trench, are the largest, pyramid-shaped papillae found at the back of the tongue (Fig. 4a). These papillae contain a plethora of taste buds, which are receptors capable of sensing five classes of taste perception: bitter, umami, sour, salty, and sweet (Fig. 4b) [76, 77]. Taste receptor cells can be categorized into three types based on functionality and morphology: Type I, II, and III, as depicted in Fig. 4c [78]. Type I cells serve as supporting cells to regulate the dynamics of neurotransmitters, particularly adenosine triphosphate (ATP) and it is also supposed to be related to the salty taste. Type II cells detect and transduce sweet, bitter, and umami stimuli, while Type III cells are sensitive for sour incentives. Type II cells are G protein–coupled receptors (GPCRs) that activate intracellular signaling pathways upon ligand binding. Most type II cells respond to only one taste quality such as bitter or sweet, because they express only one type of GPCR [75]. However, multiple taste qualities can be detected since TAS1R, TAS2R, and other taste receptors are often stimulated simultaneously. Type III cells are recognized as “presynaptic” cells with afferent nerves because they express synaptic proteins rather than GPCRs and are also thought to be involved in the detection of sour stimuli [79]. Currently, the mechanisms how the sweet, umami, and bitter taste are sensed and transduced are well investigated. These tastes share the same transduction pathway but the sweet taste is related to TAS1R2/3, bitter taste is governed by TAS2R, and umami taste is linked to TAS1R1/3 [77]. The detailed signaling cascade is as follows: the tastant binds to GPCRs, initiating a conformational change that causes the dissociation of gustducin G_α_ from G_β3_/G_γ13_. This activates phospholipase C_β2_ (PLC_β2_), leading to the hydrolysis of phosphatidylinositol 4,5-bisphosphate (PIP_2_) and the production of inositol 1,4,5-triphosphate (IP_3_) and diacylglycerol (DAG). IP_3_ promotes the opening of type 3 IP_3_ receptors (IP_3_R_3_), releasing Ca^2+^ from the endoplasmic reticulum (ER) and increasing intracellular cytosolic calcium concentration ([Ca^2+^]_i_). The elevated [Ca^2+^]_i_ activates the TRPM5 channel and the caused depolarization opens voltage-gated sodium channels to allow Na^+^ flow in and generate action potential (Fig. 4d) [79–81]. For salty taste transduction, it is believed that epithelial sodium channels (ENaCs), composed of α, β, and γ subunits, are the receptors sensitive to salt. The entry of Na^+^ through ENaCs induces depolarization, generating action potentials. However, the specific subunits involved in salty taste transduction and the complex mechanism of intracellular molecular interactions have yet to be fully elucidated. For sour taste transduction, it has been suggested that PKD2L1 and PKD1L3 are the receptors [82]. However, subsequent investigations have presented contradictory results, indicating that the ablation of these receptors does not deprive sensitivity to acids [83, 84]. Recent studies have revealed that otopetrin1 (OTOP1) is the real channel that responds to acids (Fig. 4e) [85–87]. The influx of H^+^ through the OTOP1 channel triggers depolarization and inhibits the opening of potassium (K^+^) channels (K_ir2.1_), further enhancing depolarization. Such depolarization generates action potentials by controlling the voltage-gated sodium channels.

### Olfactory Receptors

**Fig. 5:**
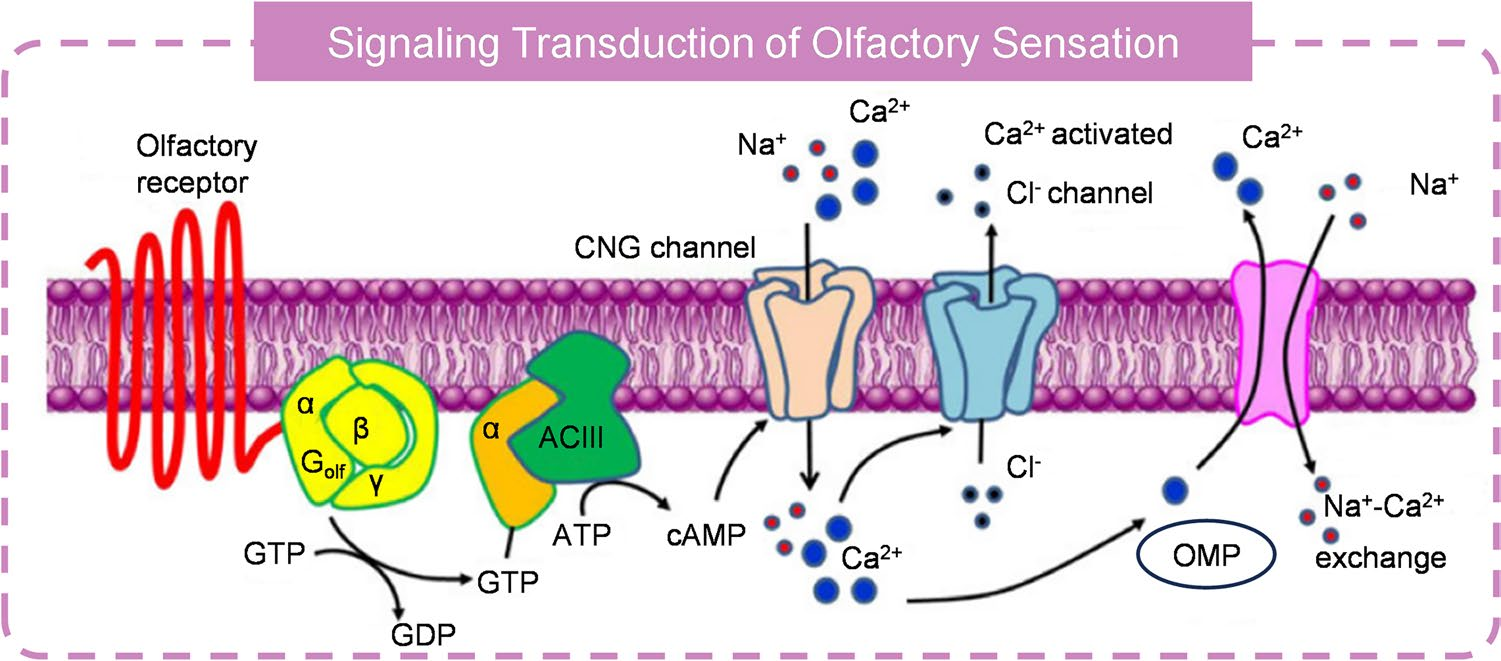
Transduction cascade of olfactory receptors. Reproduced with permission from Ref. [93]. Copyright 2012, The Korean Society for Biochemistry and Molecular Biology

The olfactory system, responsible for the sense of smell, is important for both humans and animals. It functions as a primary sensory system to detect and distinguish volatile odors in the environment. This system not only enables individuals to enjoy scents but also performs essential tasks such as identifying food, avoiding threats, and facilitating social interactions [88, 89]. For example, the ability to detect spoiled food through smell can prevent illness. Moreover, the olfactory system is closely linked to memory and emotions, with certain scents capable of evoking memories and affecting mood, underscoring its significant impact on cognitive and emotional processes. Figuring out how the nose detects odors and transduces signals into action potentials is vital for fully understanding the olfactory system. Decades of research have established that olfactory sensory neurons (OSNs) are the key cells involved in odor detection [90]. Each OSN comprises an axon, cell body, dendrites, and a dendrite knob armed by numerous cilia with hair-like structure which houses a number of odorant receptors. Olfactory transduction initiates when odorant molecules bind to these receptors (Fig. 5) [91–93]. This binding induces a conformational change and activates the trimeric, olfaction-specific G protein (G_olf_). The activated Golf stimulates the production of cyclic adenosine monophosphate (cAMP), which serves as a second messenger, from ATP by the enzyme adenylate cyclase III (ACIII). The increased cAMP level leads to the activation of a cAMP-dependent protein kinase, which phosphorylates cyclic nucleotide-gated channels (CNGCs), allowing them to open and resulting in the influx of Na^+^ and Ca^2+^ ions into the cell. Changes in intracellular and extracellular ion concentrations induces a depolarization. Moreover, the elevated Ca^2+^ levels open Ca^2+^-activated chloride ion channels (CaCCs), further depolarizing the membrane of the OSN and generating an action potential. However, the high Ca^2+^ concentration also contributes to the adaptation and recovery of the signal transduction pathway for the reason that the Ca^2+^ causes the GDP bound to the activated G_olf_ [94]. In addition, Ca^2+^ binds to Calmodulin (CaM) activates Ca^2+^ kinase type II (CaMKII). The Ca^2+^-CaM complex lowers the interaction of cAMP with CNGC, and CaMKII reduces the activity of ACIII through phosphorylation. Phosphodiesterase 1C (PDE1C) is also activated, accelerating the hydrolysis of cAMP to AMP. These processes, initiated by the influx of Ca^2+^ ions, ultimately adapt and terminate the olfactory transduction pathway.

### Auditory Receptors

**Fig. 6:**
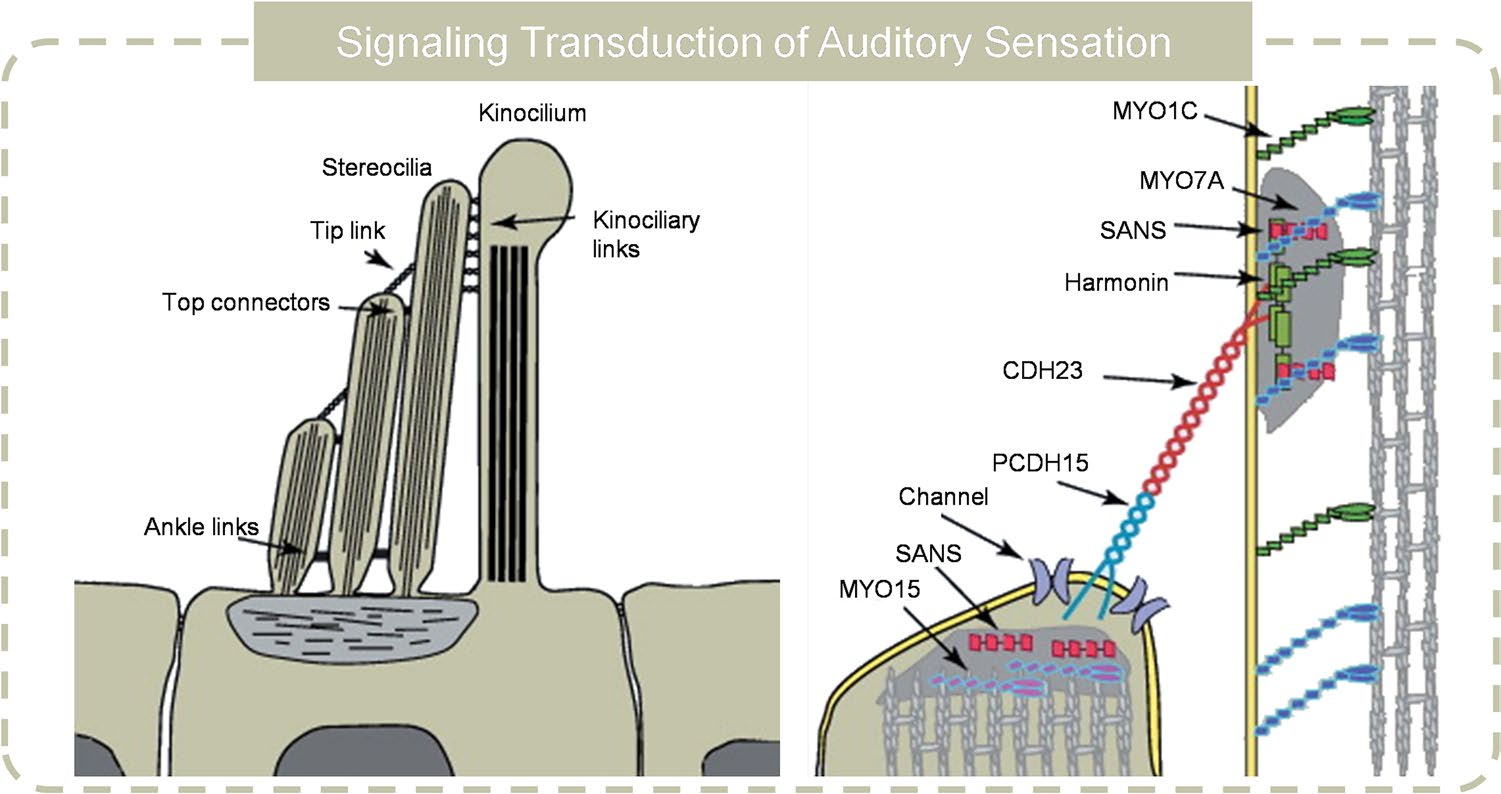
Transduction cascade of auditory receptors. Reproduced with permission from Ref. [98]. Copyright 2011, Elsevier

The ability to perceive sound is essential for communication, cognitive function, social interaction, and safety. The auditory system is the organ that detect the sound-induced vibration, amplify and convert it into electrical spikes. The auditory system has a large dynamic range, allowing to capture the sound in a large intensity and frequency range. Variations caused by sound vibration in air pressure create fluid movements in the cochlear duct, causing mechanical vibrations at the organ of Corti’s sensory epithelium [95]. The cochlea’s varying physical characteristics along its length result in frequency-specific vibrations across different segments of the epithelium. Outer hair cells (OHCs) enhance these vibrations, with the mechanical signals are then relayed to inner hair cells (IHCs) for transmission to afferent neurons [96–98]. The key process of sound perception is the auditory mechanotransduction, which is highly related to the hair bundle (Fig. 6) [98, 99]. Hair bundle is mechanically sensitive as it consists of stereocilia which arranges in an ascending way. The stereocilia are connected by the top-link filament that contains two homodimers. At the low site of the top-link filament, there are some transduction channels that can be opened by the deflection of hair bundles toward the longest stereocilia, allowing the Ca^2+^ /K^+^ flux in and cause depolarization. In addition, the ion flux triggers the adaptation, which is supposed to be beneficial for the frequency selectivity and signal amplification. Fast adaptation may occur through Ca^2+^ binding to the channel, stabilizing it in a closed state, or by Ca^2+^ binding near the channel, releasing a mechanical element that reduces tension and causes channel closure [100, 101]. Slow adaptation involves a motor protein at the upper point of top-link filament, which climbs up along F-actin to produce tension [102, 103]. Mechanical stimulation allows Ca^2+^ to enter stereocilia, which releases the motor from F-actin and closes the channel. As calcium levels drop, the motor reestablishes tension.

## Emerging Devices for ASNs

Biological sensory receptors implement the spiking encoding of external perception through intricated molecules/ions movement, enabling high energy-efficient signal processing of humans [104]. Throughout history, drawing inspiration from biology has always been an impetus to the technology advancement. These biological sensory receptors offer some clues to achieve neuromorphic sensing. Despite it is quite difficult to simulate the elaborate transduction path for perception encoding, simulating the functionality of biological sensory receptors to translate the analog signal to spikes is viable.

ASNs are devices or systems tend to mimic the behavior of biological sensory neuron. As mentioned above, it commonly consists of diverse kinds of sensors to detect the stimuli and artificial neuronal devices to produce spikes to encode sensory data. To overcome the area and energy disadvantage of CMOS-based artificial neuronal device, up to date, various innovative memory devices for artificial neuronal devices have been developed, including single transistors, 2D memtransistor, memristors, phase change memory, magnetic tunneling junctions, and ferroelectric memory [105–112]. Below we’ll highlight emerging devices that have been employed to implement ASN.

### Memristor

Memrisive devices have been widely used for neuromorphic computing [17, 113, 114]. The diffusive memristor or redox-based memristor, is an electronic device that exhibits a reversible change in resistance under electric field effect [115, 116]. The dielectric materials typically used in diffusive memristor include metal oxides, conducting polymers, and solid electrolytes, while the electrodes generally are active metals [117]. The working mechanism of a diffusive memristor involves the migration of charged ions or vacancies within the material when a sufficient electric field is established [118, 119]. When a voltage is applied across the memristor, the electric field causes ions to move from the metal electrode into the oxide, creating a conductive path known as a filament. As the ions accumulate, the filament grows, and the resistance across the memristor decreases, allowing more current to flow. Conversely, when the electric field is reversed, the ions begin to diffuse back toward the original electrode, shrinking the filament and increasing the resistance. This bidirectional adjustment of the filament’s size and the corresponding resistance change is the essence of the operation of the diffusive memristor. The resistance change of the memristor is tunable by controlling the voltage magnitude and polarity [120–122]. Based on the working mechanism of the diffusive memristor, the electrodes and insulating layer are required to pose some specific characteristics to contribute to the formation/rupture of filaments. The active metals should be easily ionized and diffused into the insulating layer. In addition, the metal element will undergo redox reactions under the electrical field. For the insulating layer, the materials should be chemically stable and have some degree of ionic conductivity rather than electrically conductive.

**Fig. 7:**
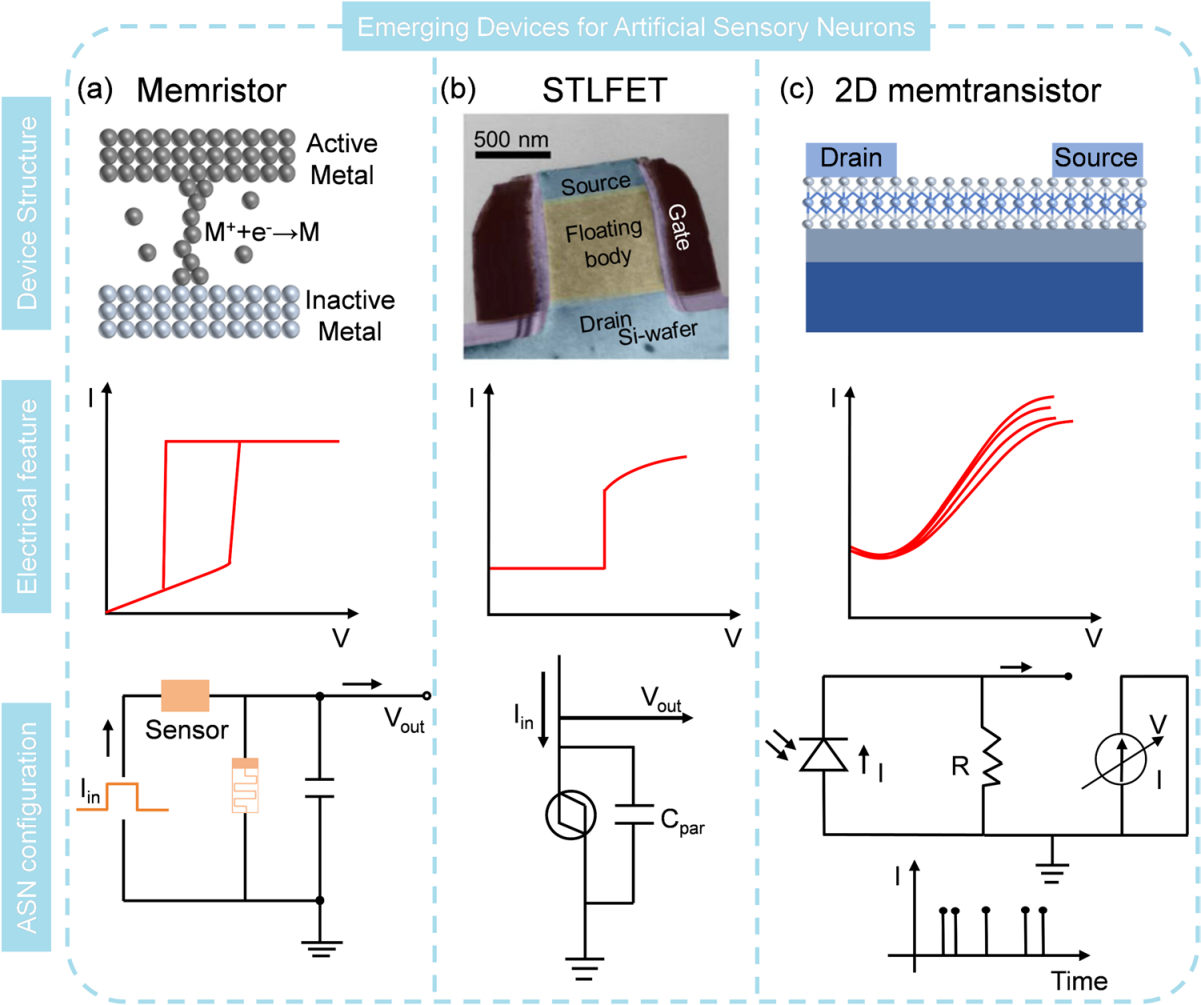
Structure, electrical characteristics and ASN implementation circuit of **a** memristor, **b** STLFET, Reproduced with permission from Ref. [142]. Copyright 2020, American Chemical Society and **c** 2D memtransistor

The Mott memristor, another type of memristor, also exhibits a reversible change in resistance based on the Mott transition, a quantum mechanical effect observed in certain materials, such as transition metal oxides like vanadium dioxide (VO_2_) and niobium oxide (NbO_x_) [123–126]. These materials undergo a phase transition from a semiconducting state (HRS) to a metallic state (LRS) when an electric field or temperature is applied. In detail, in its initial state, the oxide acts as an insulator due to the localization of electrons, which are trapped in orbitals around the metal ions. When a sufficiently high voltage is applied, the electric field causes the electrons to delocalize, transitioning the material into a conductive state known as a Mott insulator. This transition is accompanied by a significant drop in resistance, allowing current to flow through the material. Removing the electric field allows the material to return to its insulating state as the electrons re-localize, restoring the original high resistance [127–129]. There are some key features of Mott memristive materials. First, Mott materials must have fast and reversible switching capabilities, enabling the memristor to switch between states rapidly. Second, they need to exhibit high thermal stability and robustness to fatigue to guarantee that there is no obvious performance degradation of switching cycling. Both diffusive and Mott memristors can be harnessed for ASN by integrating them with external resistor, capacitor, and various sensors (Fig. 7a). When the applied voltage reaches the threshold voltage, the memristor transits to LRS, leading to a rapid discharge of a capacitor, which in turn reduces the applied voltage. However, when the voltage falls below the holding voltage, the memristor spontaneously returns to its HRS, terminating the discharging process and allowing for recharging [130, 131]. In this process, a periodic increase and decrease in output voltage/current can be observed and the oscillatory frequency is strongly dependent on the circuit’s charging and discharging speeds.

The performance of ASN based on diffusive and Mott memristors differs significantly. Diffusive memristors typically use a leaky integrate and fire (LIF) strategy for spike generation with a relatively long integration time that results in a spike frequency ranging from 1 to 1000 Hz [132, 133]. In addition, the ultralow current of diffusive memristors allows for low power operation, but their large variation need to be further improved. Mott memristors, on the other hand, show high uniformity after device optimization and have been used for hardware verification of SNN. They usually employ an oscillation method for spike generation, which can produce spike frequencies up to kHz or MHz, making them suitable for fast encoding and processing [134–136]. However, the output current of Mott-based ASN is typically in the milliampere range, leading to higher energy consumption.

### Single Transistor Latch-Based Field Effect Transistor (STLFET)

Recently, ASNs based on the single transistor latch (STL) phenomenon have been reported. Their structures are planar or vertical n-p-n devices with a floating p-based region. The STL effect, which occurs at high drain biases, is an extreme case of floating-body effects observed in silicon-on-insulator metal–oxide–semiconductor field-effect transistors (SOI MOSFETs) [137, 138]. When the drain bias is high, the produced impact ionization current close to the drain forward biases the body-to-source diode, which raises the body potential. This enhanced body bias subsequently lowers the threshold voltage of the SOI MOSFET, causing an upsurge in the drain-to-source current and generating more impact ionization current. This positive-feedback phenomenon, which happens when the impact ionization current surpasses the leakage current of the body-to-drain diode, triggers a sudden increase in the subthreshold current and the body potential. However, this positive feedback has its own limitation: as the body bias increases, the drain saturation voltage increases accordingly, leading to a reduction in the channel electric field and a decrease in the impact ionization current. Furthermore, as the drain current intensifies, the effective potential across the channel reduces because of the resistance drop in the source and drain regions. To achieve the STLFET, the materials characteristics should be carefully considered. High electron mobility of semiconducting channel is desirable. Besides, high dielectric constant of gate provides precise control over the channel and the source and drain materials must form a ohmic contact with channel to allow current flow. The STL phenomenon can be exploited for spike generation through circuit design (Fig. 7b) [139–141]. When a constant input current (I_in_) is applied to the collector, positive charges accumulate in the parasitic capacitance because the STLFET is in HRS when a low voltage is applied. With the accumulated positive charges, the output voltage (V_out_) measured at the collector increases. When V_out_ exceeds the latch-up voltage (V_latch_), the accumulated charges flow out toward the emitter to decrease V_out_, which is the firing process as the STLFET changes to LRS. After firing, the STLFET automatically returns to the HRS to enable repetitive integrations in the resting state. STL-based ASNs offer several advantages: i)their CMOS-compatible manufacturing enables excellent integration with other circuit components and high uniformity/stability, which is beneficial for the mass fabrication of ASN; ii) the driving current is very low (~ nA), showing great potential for low-power applications. The generated spike frequency of STL-based ASNs is less than 1 kHz because the charging and discharging time is roughly at millisecond level.

### 2D Memtransistor

**Fig. 8:**
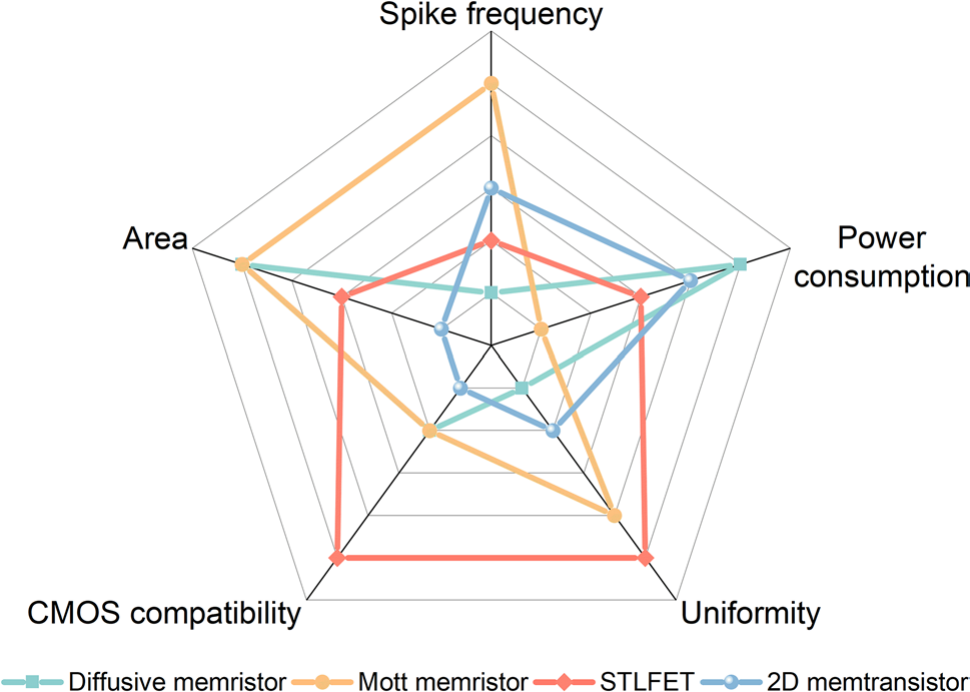
Performance comparison of diffusive memristor, Mott memristor, STLFET, and 2D memtransistor in terms of area, CMOS compatibility, uniformity, power consumption, and spike frequency

A memtransistor, also known as a memory transistor, is a cutting-edge device that merges the functionalities of a transistor and a memory [143–145]. It features three terminals: two electrodes connected with atomically thin channel and a third gate that modulates the electrical behavior. Memtransistors commonly rely on Schottky barrier tuning such as vacancy/ion transport, charge trapping, or ferroelectric domain switching at metal–semiconductor interfaces. The working mechanism involves the use of an external gate voltage to modulate the carrier density in the 2D material, controlling the transistor’s channel conductance. Additionally, the application of a suitable bias across the source and drain electrodes induces a non-volatile change in the resistance state of the 2D material, akin to a memristor, which is achieved through the movement of defects, ions, or by altering the material’s lattice structure. This dynamic resistance can be "remembered" even after the voltage is removed, providing non-volatile memory storage. The transistor-like functionality allows for the modulation of current between the source and drain terminals, making the device reconfigurable for advanced neuromorphic computing [146–149]. To fabricate the 2D memtransistors, the 2D semiconducting materials should be atomically thin with high electron mobility. Additionally, the bandgap of 2D materials is preferably tunable to regulate the resistance of memtransistors. Similar to the STLFET, the electrodes need to make a low-resistance ohmic contact with 2D materials. The current–voltage characteristics of memtransistors demonstrate a significant increase in current level with increasing gate voltage. When the current surpasses a predefined current threshold, a current spike can be detected. Leveraging this characteristic, memtransistors can be identified as ASNs alone or by connecting it with other sensors to implement ASN (Fig. 7c). External stimuli can directly alter the current behavior or indirectly influence it by adjusting the gate voltage, thereby controlling the spike dynamics. The spike frequency of memtransistor-based ASN is typically around kHz, which is higher than that of diffusive memristor and STLFET-based ASN. Two-dimensional (2D) semiconductors, such as molybdenum disulfide (MoS_2_) and indium selenide (In_2_Se_3_), have been extensively explored for memtransistor applications [146, 150–154]. Harnessing the unique properties of 2D materials such as high surface-to-volume ratio, the memtransistor shows superior sensing abilities, which are beneficial to enhance the sensitivity. The performance comparison of diffusive memristor, Mott memristor, STLFET, and 2D memtransistor is shown in Fig. 8 and detailed characteristics of these devices are listed in Table 1.

**Table 1:** Detailed characteristics of diffusive memristor, Mott memristor, STLFET, and 2D memtransistor

Device	Diffusive memristor	Mott memristor	STLFET	2D memtransistor
Working mechanism	Redox reaction	Phase change	Impact ionization current modulation	Carrier modulation
Advantages	Compact structure, low power	Compact structure, high uniformity	CMOS compatibility, low power consumption, high uniformity	High tunability
Limitations	Large cycle-to-cycle and device-to-device variation	High power consumption,	Large area	Large area, large variation
Materials characteristics	Active electrode such as Ag, Cu and chemical stable dielectric layer	Mott materials that can switch between insulating and conducting states due to phase transition	Semiconductor channel with high carrier mobility and gate with high dielectric constant	2D semiconductor materials (e.g., MoS₂, graphene) with atomic thinness, high mobility, and tunable electronic properties
Functionality	Volatile memory	Volatile memory	Volatile memory	Non-volatile memory

## Various Types of ASN

### Artificial Tactile Neuron (ATTN)

**Fig. 9:**
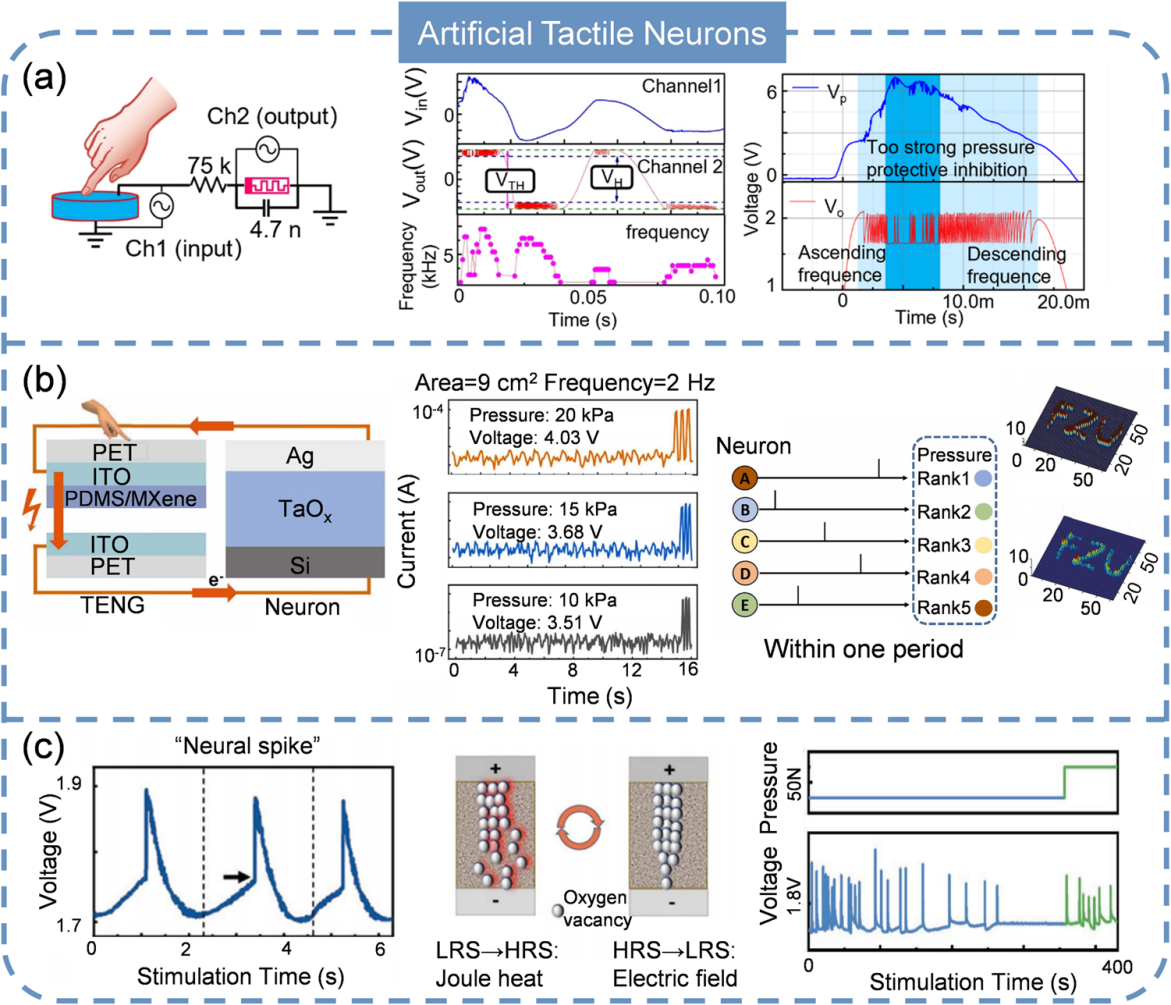
Artificial tactile neurons. **a** ATTN with Mott memristor. Reproduced with permission from Ref. [159]. Copyright 2020, Springer Nature. **b** ATTN with diffusive memristor and rank order coding algorism. Reproduced with permission from Ref. [165]. Copyright 2022, Elsevier. **c** ATTN with biologically neuronal adaptation. Reproduced with permission from Ref. [177]. Copyright 2022, John Wiley & Sons

Artificial tactile systems are engineered to mimic or augment the sense of touch in artificial or robotic entities. These systems find extensive application across diverse fields, including robotics, prosthetics, virtual reality, and medical devices [155–158]. The stringent criteria for power efficiency and footprint in these applications present challenges that traditional sensors often struggle to meet. ATTN, which imitate the characteristics of biological tactile systems, represent a promising solution to address these challenges. They are capable of detecting pressure cues and converting them instantaneously into spikes for subsequent processing. At present, Mott memristors are the most widely used devices to achieve tactile encoding. For example, Zhang and colleagues conducted groundbreaking work on ATTN by integrating a NbO_x_ memristor with a piezoelectric device (Fig. 9a) [159]. In this configuration, the piezoelectric device functions as a self-powered source, converting tactile signals into electrical voltage. It generates voltage at the onset of pressure, reaching a maximum output that corresponds to the degree of exerted pressure and subsequently decays due to the charge leakage. The frequency of output spikes is sensitive to the amplitude of the source voltage. Importantly, when the pressure is high, the produced voltage is also high, suppressing the ATTN to generate spikes, which is analogous to the biological protective inhibition observed in living organisms. This work demonstrates the viability of using NbO_x_ memristors to transform tactile information into spikes by establishing a correlation among pressure, input voltage, and spiking rate. Despite progress, this work lacks the ability of resembling to the behavior of biological mechanoreceptors such as low adaptive receptors (SA), which response to sustained static pressure. In addition, the spatial integration of tactile perception via ATTN, which combines sensory data from various tactile receptors to create a comprehensive understanding of the spatial characteristics of objects and the environment, has not been reported.

With these challenges in mind, Li et al. [160] fuses a NbO_x_-based Mott memristor with a polypyrrole (PPy)-based resistive pressure sensor with micro-pyramidal structures. The spike generation frequency shows a linear relationship with the applied pressure stimuli. Notably, the ATTN operates at an exceptionally low range (below 1 kPa), enabling the detection and conversion of slight pressures. To illustrate the concept of spatial tactile integration, two pressure sensors were connected to the memristor. The results indicated that the integrated spiking frequency from parallel sensors was higher than that from individual sensors, implying a significant reduction in response time and an improved speed of tactile perception. Integrating the ATTN with other hardware is important for tasks like recognition, Wen et al. [161] developed an artificial perceptual learning system capable of encoding and decoding Morse code. The system’s core components include a ATTN with a perception module, an encoding module, and a microcontroller unit (MCU). The ATTN converts the pressure into spikes and the MCU counts these spikes, decodes them, and displays the Morse code translations through an array of LED lights. This method has been verified by successfully encoding and interpreting all 26 letters of the English alphabet.

Unfortunately, a single device is difficult to meet the requirements of practical applications where multiple sensors should be deployed. A sensor array can significantly enhance detection capabilities because the combined response of multiple sensors provides more comprehensive information than a single sensor could [162]. Additionally, using an array can improve overall system reliability by reducing the risk of complete failure, and mitigating the potential for errors or malfunctions that could be caused by a single sensor [163]. Fang et al. [164] reported a 3 × 3 ATTN array with VO_2_ memristors and resistive sensors. Such array can be utilized to realize letter recognition by coupling it with a voltage comparator and a field-programmable gate array (FPGA). When various stimulus patterns (~ 5 kPa) are applied to the 3 × 3 array, the resultant output spikes are processed and subsequently visualized on a screen. Moreover, a larger 64 × 64 ATTN array was proposed by Ye and colleagues through a triboelectric nanogenerator (TENG) and diffusive memristor (Fig. 9b) [165]. With this array, the trajectory recognition/texture extraction were completed. The ATTNs detect the signal and generate spikes when pressure is applied, distinguishing the pressured units from those unpressurized. Note that the temporal information of spiking reflects the strength of pressure, therefore, the pressing intensity of each cell can be extracted by decoding the first spiking timing based on rank order coding algorism (rank order coding refers to a mechanism by which information is encoded in the relative timing or frequency of neuronal spiking rather than the absolute timing or frequency) [166, 167].

For the envisioned applications in robotics, neural prosthetics, and skin electronics, it is essential that ASNs are capable of being seamlessly integrated onto curved surfaces or of being worn on the body [168–170]. Developing flexible ASNs is indispensable since ASN mounted on rigid substrates are not well-suited for these applications due to their inability to conform to uneven shape. Recently, a flexible ATTN was proposed with a VO_2_ memristor and a resistive sensor [171]. The spiking performance of the flexible ATTN remains stable even after 1000 bending cycles. Leveraging this robust stability, a flexible tactile encoding system designed for motion direction recognition has been demonstrated. In this system, three distinct input voltages are applied to three separated ATTNs. By analyzing the frequencies of the output spikes in the temporal domain, the direction of movement can be accurately distinguished. This demonstrates the system’s capability to interpret complex tactile information and could have significant implications for applications in wearable technology, human–machine interfaces, and smart prosthetics.

The mass production of ATTNs is not feasible without relying on sophisticated semiconductor fabrication techniques. While Mott memristors are widely utilized due to their high uniformity and stability, the large-scale manufacturing presents significant challenges. Consequently, there is an immediate need to investigate and develop new ATTNs that are compatible with established foundry processes. Based on STLFET and a TENG, Han et al. [172] reported an ATTN with ~ kHz spike frequency. The key fabrication process of STLFET entails arsenic doping for the emitter and collector and boron doping for the base with all dopants activated by subsequent rapid thermal annealing. These steps are compatible with current CMOS technology, which means that the STLFET can not only be mass-produced but also can be seamlessly integrated with peripheral circuits to form complex sensory systems. This compatibility simplifies the design and manufacturing process, as engineers can utilize familiar tools and techniques while exploiting the unique features of the STLFET-based ATTNs.

In the field of biology, neuronal adaptation, also known as firing rate adaptation, is a fundamental property of neurons that allows them to modify their response to a constant or repetitive stimulus [173, 174]. Mechanoreceptors with fast adaptation, including Meissner’s corpuscles and Pacinian corpuscles, promptly react to variations in mechanical inputs such as touch and vibration, yet they quickly reduce their activity when the stimulation keeps constant [175]. This enables the nervous system to focus on the perception of dynamic alterations rather than the sustained pressure. For instance, upon contacting with a surface or gripping an item, these receptors are instantly activated to convey the initial sensation and the surface’s texture. Nevertheless, once the contact remains unaltered, their signaling diminishes, enabling you to disregard the ongoing tactile sensation and instead respond only to new events, such as the object’s movement or a shift in its position. This fast adaptation is vital for activities that demand intricate motor skills, like manipulating objects or discerning minute variations on surfaces. While mechanoreceptors with slow adaptation, such as Merkel cells and Ruffini endings, persistently firing in the presence of ongoing mechanical stimulation, which allows the nervous system to monitor continuous pressure or deformation over an extended period [176]. In contrast to the fast adaptation, these receptors provide continuous response about the stimulus’ intensity and duration, rendering them indispensable for detecting steady applied forces, such as holding an object. For example, when you grasp a cup, the slow-adapting receptors consistently detect the pressure the cup exerts on your skin, guaranteeing that you sustain an appropriate hold. Such sustained response is crucial for tasks that require prolonged attention to pressure and force, such as holding or manipulating objects without dropping them. These behaviors enable receptors to adjust their firing patterns, which helps change the sensitivity of neurons, expand the dynamic range, maintain precise motor control, filter out background noise, and focus on meaningful or significant stimuli, which is essential for processing complex information. Adopting the ionic memristor with Pt/Co_3_O_4-x_ /ITO structure, Xie et al. [177] realized an ATTN that was capable of detecting slight pressure changes under the background of constant strong pressure to emulate the tactile adaptation characteristics of human skin (Fig. 9c). When a force is constantly exerted, a spiking adaptation can be observed with decreased spike frequency. Interestingly, a slight increase in current amplitude terminates the adaptation and restores its initial spiking frequency, enabling the capture of subtle pressure fluctuations in the environment. However, developing ATTNs that can simultaneously mimic the slow adaptation and fast adaptation of mechanoreceptors is still challenging probably because the tunability of firing dynamics of single ATTN is not easy. A reconfigurable ATTN is imperative to achieve multimodal perception, not only for tactile, but also for visual, olfactory and other senses.

### Artificial Thermal Neuron (ATMN)

**Fig. 10:**
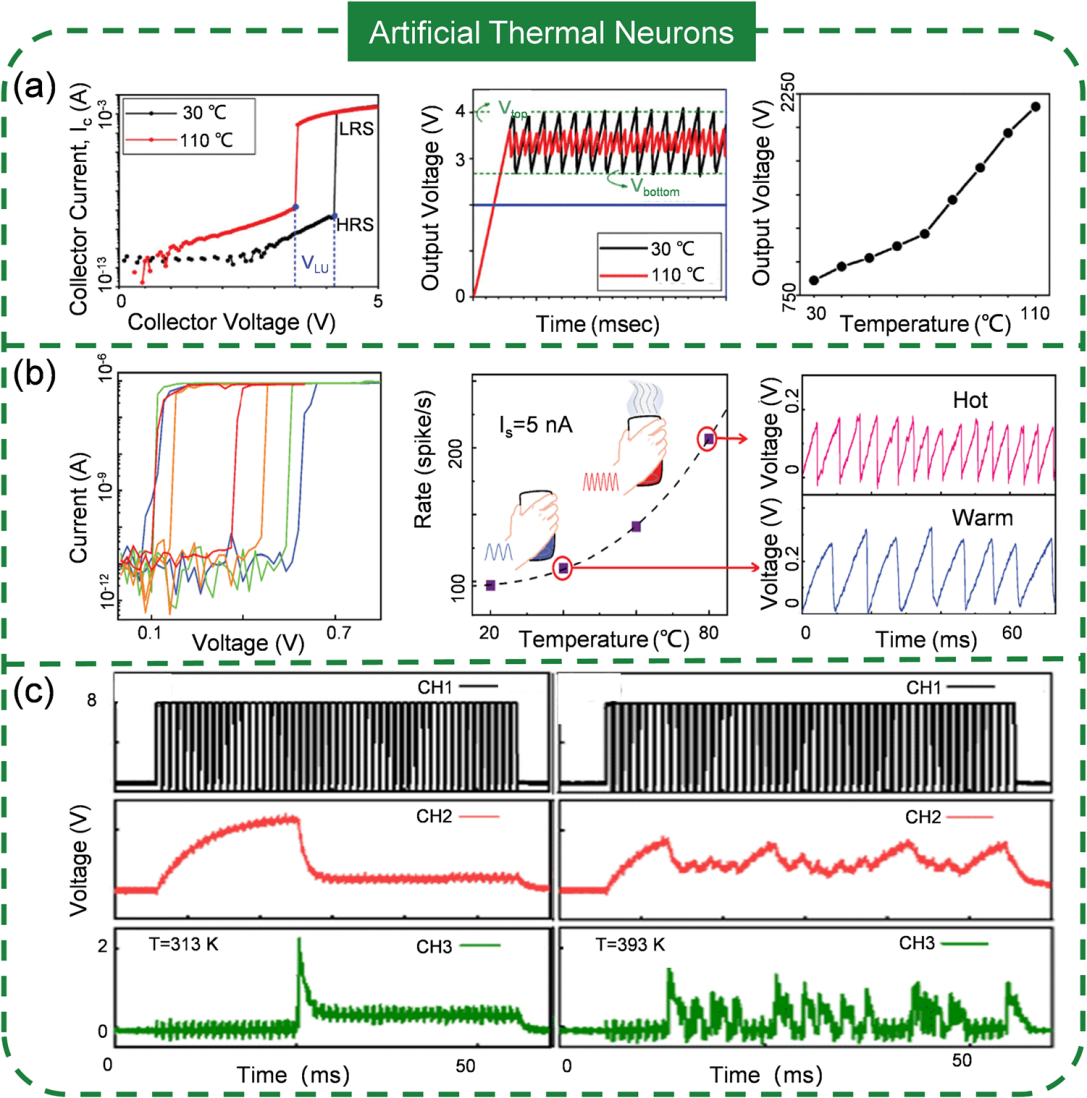
Artificial thermal neurons with various emerging devices: **a** STLFET; Reproduced with permission from Ref. [178]. Copyright 2021, IEEE. **b** Diffusive memristor; Reproduced with permission from Ref. [180]. Copyright 2022, IEEE. **c** Mott memristor. Reproduced with permission from Ref. [182]. Copyright 2023, Royal Society of Chemistry

Upon contact with an object, we perceive not only its pressure but also its temperature, which informs us of its current state. Consequently, it can be inferred that ATMNs are integral to constructing artificial perception systems. The emerging devices employed for ATTNs also can be hired for ATMNs, albeit with distinct implementation configurations. Because of the inherent thermal effect of these devices, they can act as thermal sensors by themselves. Therefore, the circuit for completing the ATMNs is simpler compared to other classes. Lee et al. [178] have recently developed an ATMN with a STLFET whose electrical characteristics, specifically its threshold voltage, are strongly influenced by temperature (Fig. 10a). As temperature rising, the threshold voltage decreases, leading to the spiking frequency increase. This is attributed to the higher temperature increasing the ionization rate, permitting more holes to accumulate in the base region, which lowers the transistor’s threshold voltage. Detailed analysis of the spiking frequency across different temperatures further supports this mechanism. The thermal encoding operation range of ATMN is 30 ~ 110 °C, offering a broad dynamic range suitable for various applications in IoT. Similarly, a temperature-regulated ATMN using a diffusive memristor is reported by Wu and colleagues [179]. The temperature dominates the Ag filament formation by controlling the activation energy for Ag ion transport. Higher temperatures enable the formation of a conductive channel at a lower electric field, leading to an increased spiking frequency and a reduced delay time. However, challenges such as high-power consumption (up to microjoules) remain to be resolved. Shi et al. [180] have endeavored to improve these by optimizing the device’s structure, extending its range from 20 to 80 °C and reducing power consumption to be as low as 90 pJ spike^−1^ (Fig. 10b). An AlO_x_ insulating layer is inserted into the device to mitigate leakage current, thus enabling low energy consumption. Despite these advancements, the inherent variability of Ag-based artificial neuronal devices still poses stability challenges, with higher temperatures potentially exacerbating fluctuations in temperature encoding. Mott insulator is another candidate that can be applied to the ATMNs. Han et al. [181] introduced a flexible ATMN utilizing VO_2_, which operates from 25 °C (with a spiking frequency of 16.9 kHz) to 40 °C (60.2 kHz). They developed a physical model based on 3D Pool–Frenkel emission and Newton’s law of cooling, distinct from those of diffusive memristor-based or STLFET-based ASN. The model’s simulation results align well with experimental data in the range of 20 to 40 °C. This design holds promise for integration into flexible neuromorphic intelligent systems. In addition, Zhao et al. [182] reported an ATMN with a working range from 40 to 120 °C using NbO_x_ (Fig. 10c). For Mott materials, the temperature sensing capability is determined by the IMT temperature. Therefore, the NbO_x_-based sensor offers a broader detection range than the VO_2_-based sensor, as the IMT temperature of NbO_x_ is higher (800 °C) compared to VO_2_ (67 °C). While the progress in temperature encoding is encouraging, some considerations must be figured out. Many reported neuromorphic temperature sensors consume high energy, making them less competitive with biological temperature receptors. Additionally, there is a need for sensors that can encode an ultra-wide temperature range, such as from 0 to 100 °C or beyond, which probably can be addressed through material and device engineering advancements.

### Artificial Acoustic Neuron (AAN)

**Fig. 11:**
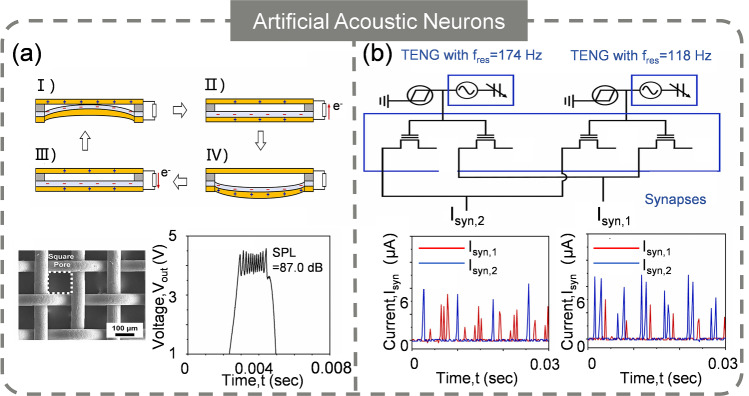
Artificial acoustic neurons. **a** Working mechanism of acoustic sensor. **b** Hardware implementation of acoustic differentiation. **a**–**b** Reproduced with permission from Ref. [187]. Copyright 2023, Elsevier

Artificial acoustic systems are devised to replicate, enhance, or modify sound for various applications such as cochlear implants and robotic navigation, perception, localization, and interaction [183, 184]. They include loudspeakers, microphones, soundbars, and home theater systems, which are used to produce and capture sound for entertainment, communication, and recording purposes. Nevertheless, these systems are bulky and high-power-consuming because of the complex circuit implementation [185, 186]. AANs offer a more efficient alternative by mimicking the human auditory system’s ability to convert sound waves into spikes, which is compact and consumes significantly less energy. Yun et al. [187] suggested an AAN by merging a TENG that is responsive to sound pressure with STLFET. The TENG is structured with a top copper electrode, a fluorinated ethylene propylene (FEP) film bonded to a base copper electrode (FEP/Cu), and eight spacers to keep the top electrode and FEP film apart. Sound wave pressure on the FEP/Cu through a mesh causes it to vibrate, generating alternating current via triboelectrification (Fig. 11a). This current makes the STLFET oscillate, and louder sounds produce more spikes. An artificial acoustic module has also been developed for pitch classification, with two neuron modules resonating at 118 and 174 Hz used to classify C3 (130.8 Hz) and G3 (196.0 Hz) piano pitches (Fig. 11b). The input layer had neuron modules with TENGs of 118 and 174 Hz, and the output layer had two synaptic nodes: one for C3 and one for G3. With a winner-take-all principle, the system identified pitches by comparing synaptic frequencies. This is the first attempt to develop AAN inspired by biological auditory neural pathways.

### Artificial Gustatory Neuron (AGN)

**Fig. 12:**
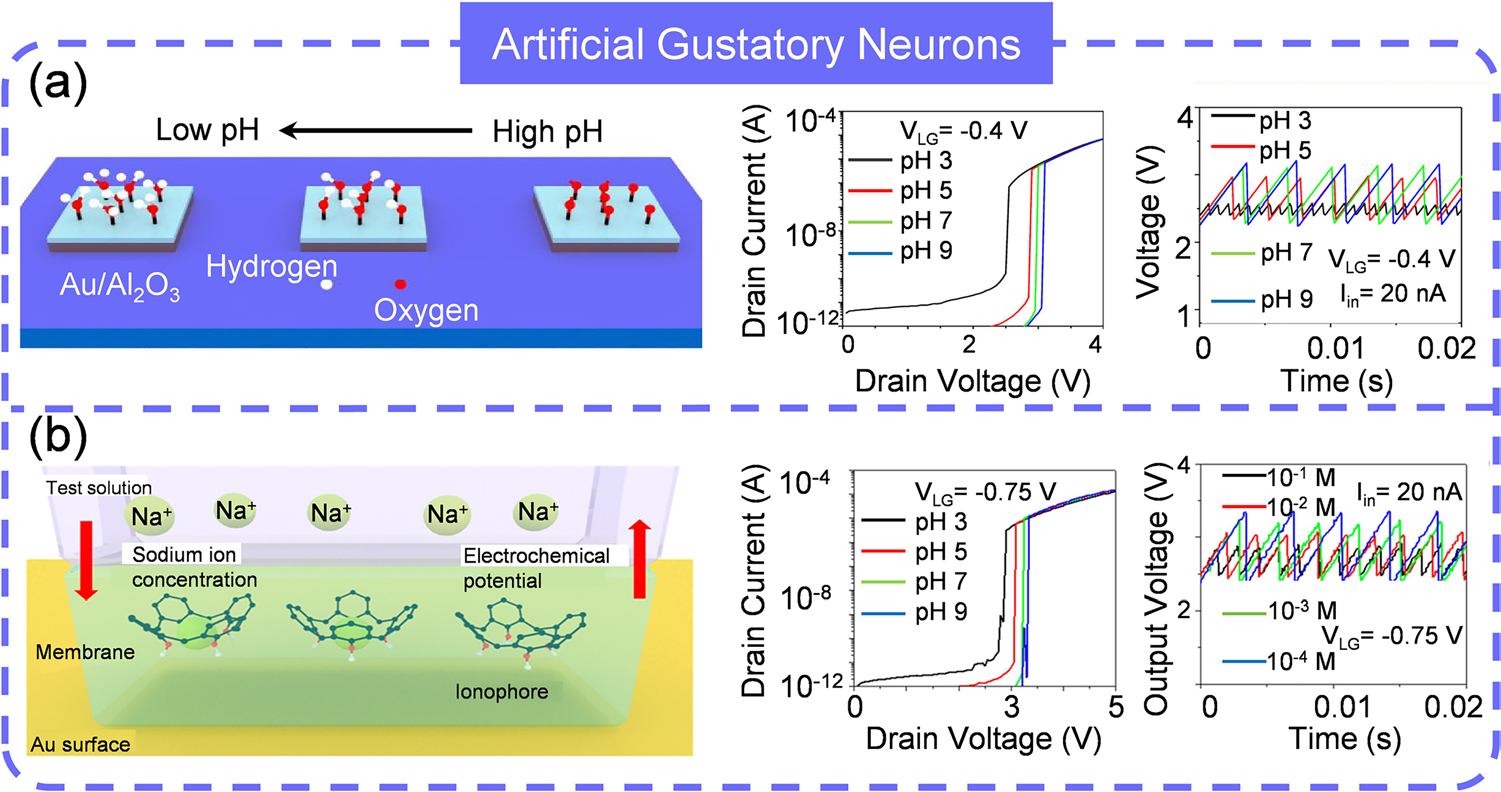
Artificial gustatory neurons. **a** AAN for pH sensing. **b** AAN for sodium ion sensing. **a**–**b** Reproduced with permission from Ref. [191]. Copyright 2022, American Chemical Society

Electronic tongues mimic the human sense of taste. Utilizing arrays of chemical sensors, it can be employed in the food and beverage industries to analyze and classify tastes [188–190]. By interpreting output signals through pattern recognition algorithms, they discriminate and quantify various taste sensations. Recently, an AGN, which composes of a pH sensors/sodium ion sensor and STLFET, has been introduced by mimicking the biological taste receptor [191]. As the pH decreases, the buffer solution produces more hydrogen ions that bind to the hydroxyl groups on the Al_2_O_3_ surface, resulting in an increase in both surface charge and potential. This higher surface charge and potential decrease the threshold voltage of the STLFET, reducing the energy barrier and increasing the oscillation spiking frequency. Hence, low pH values can be encoded by high output spiking frequencies, and vice versa (Fig. 12a). Similarly, a sodium-sensitive sensor made of sodium ionophore X can represent low or high sodium ion concentrations by high or low output spiking frequencies. The sodium-selective ionophore generates a membrane potential by capturing sodium ions, driven by the concentration gradient across the membrane and solution interface (Fig. 12b). Furthermore, a neuromorphic E-tongue capable of distinguishing between different flavors, such as vinegar and brine, has been proposed. The tastes are effectively separated based on the spike dynamics of the two output nodes. This work demonstrates the advantages of spiking gustatory architecture over traditional systems in terms of energy efficiency and hardware cost. However, significant effort is still required to advance its practical application. The human sense of taste can detect five primary tastes including sweet, sour, salty, bitter, and umami while the spiking gustatory system that accomplish the encoding of sweet, bitter, umami is lacking. Integrating all taste encoding abilities into a single system, akin to the human tongue, is essential for interaction with other electronic systems. An evident concern is the decoding of spike signals. When multiple tastes are detected simultaneously by the all-in-one system, correctly decoupling the output spikes is crucial for accurate interpretation, avoiding errors or omissions. Analyzing spike amplitude or frequency with algorithms may be beneficial, and establish a one-to-one correspondence between spike frequency and input flavors by regulating the electrical performance of AAN is another option.

### Artificial Olfactory Neuron (AON)

**Fig. 13:**
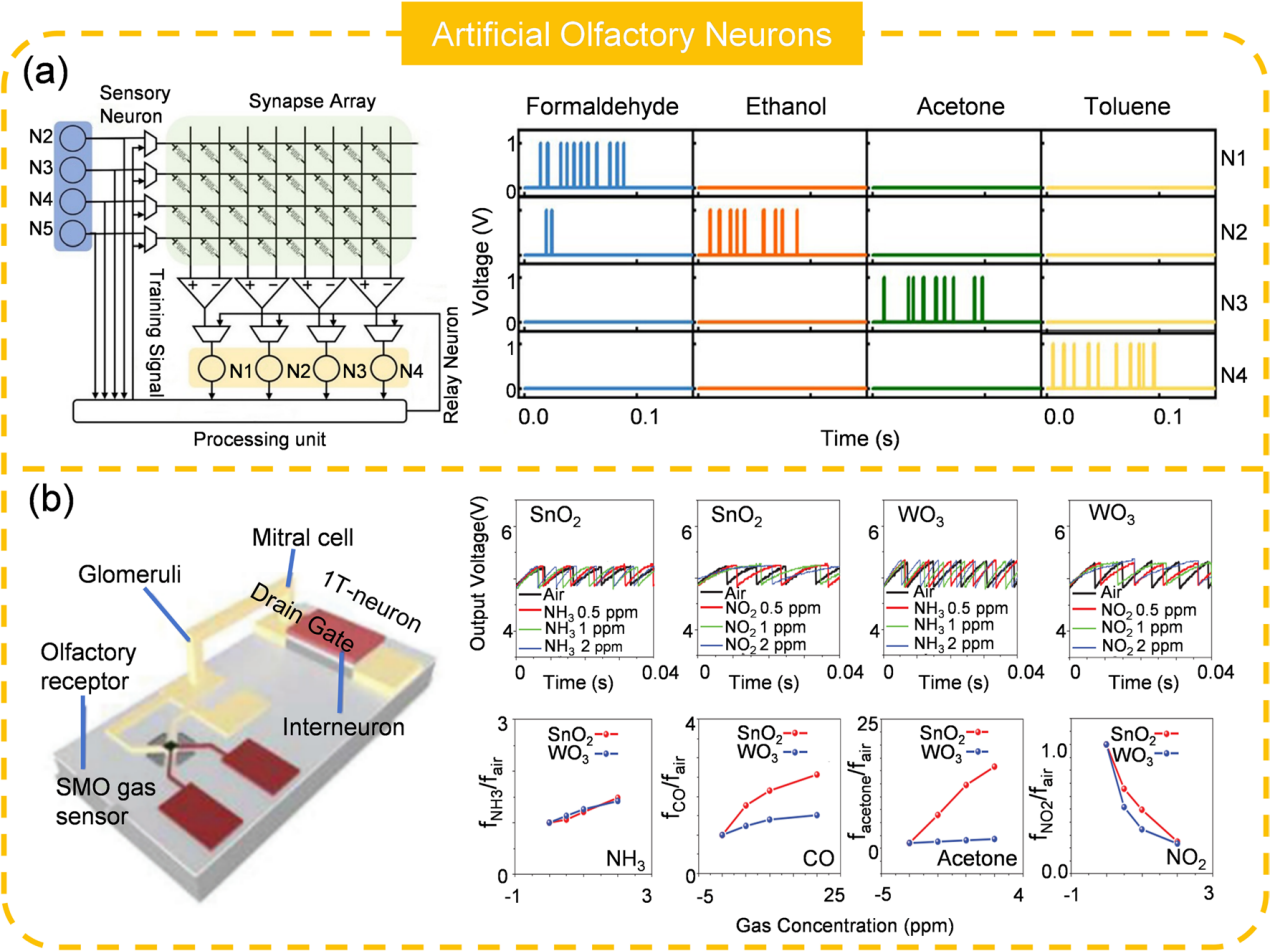
Artificial olfactory neurons. **a** AON-based neural network for gas recognition. Reproduced with permission from Ref. [197]. Copyright 2022, John Wiley & Sons. **b** AON for gas type and concentration detection. Reproduced with permission from Ref. [198]. Copyright 2022, John Wiley & Sons

Artificial olfactory system, also known as electronic noses or e-noses, are apparatus that imitate the function of the human olfactory system, detecting and identifying odors [192, 193]. These devices can detect volatile organic compounds and other chemical substances in the air, and are extensively applied in a variety of applications such as food quality, environmental monitoring, and diagnostics [194–196]. Most of the existing olfactory systems use traditional architecture, which impose severe demands on the transmission bandwidth and subsequent computational resources. To tackle this issue, Wang and colleagues developed an artificial olfactory system that integrates gas sensing, data storage, and processing functions (Fig. 13a) [197]. The system’s encoding units are realized by wiring the commercial gas sensors to a diffusive memristor. The synapses, based on nonvolatile memristive devices, transmit signals from AON to relay neurons according to synaptic weights that are trained through supervised spike-rate dependent plasticity (SRDP). The relay neurons process the signals from the synapses and classify the gases. A processing unit, organized with a field-programmable gate array (FPGA) and an application-specific integrated circuit (ASIC) for signal processing and generation, receives the outputs from the neurons and distributes the necessary control signals to the respective components. This olfactory system can clearly identify four gas samples (formaldehyde, ethanol, acetone, and toluene) with different spiking patterns. However, the system does not encode gas concentration, which is an essential dimension of odor information. Han et al. [198] reported an AON that can convert both the gas type and the concentration to spikes, significantly expanding its applicability in areas such as air quality and toxicology monitoring (Fig. 13b). The AON contains a semiconductor metal oxide (SMO) sensor and a STLFET. It is important to note that the gas absorption reactions on SMO sensors are typically activated at higher temperatures (200 to 400 °C), necessitating a microheater. With SnO_2_-based and WO_3_-based sensors, the AON can implement the spiking encoding of gas species (NH_3_, CO, acetone, NO_2_) with different concentrations. However, an encoding problem exists in such systems. For instance, 2 ppm of NH_3_ and 20 ppm of CO can share a very similar spiking frequency with the neuromorphic nose module composed of the WO_3_ gas sensor and the MOSFET neuron. Furthermore, the inclusion of the microheater significantly increases the power consumption of the entire system. Further development of novel gas sensors or AONs will assist to mitigate these dilemmas.

### Artificial Biochemical Neuron (ABCN)

**Fig. 14:**
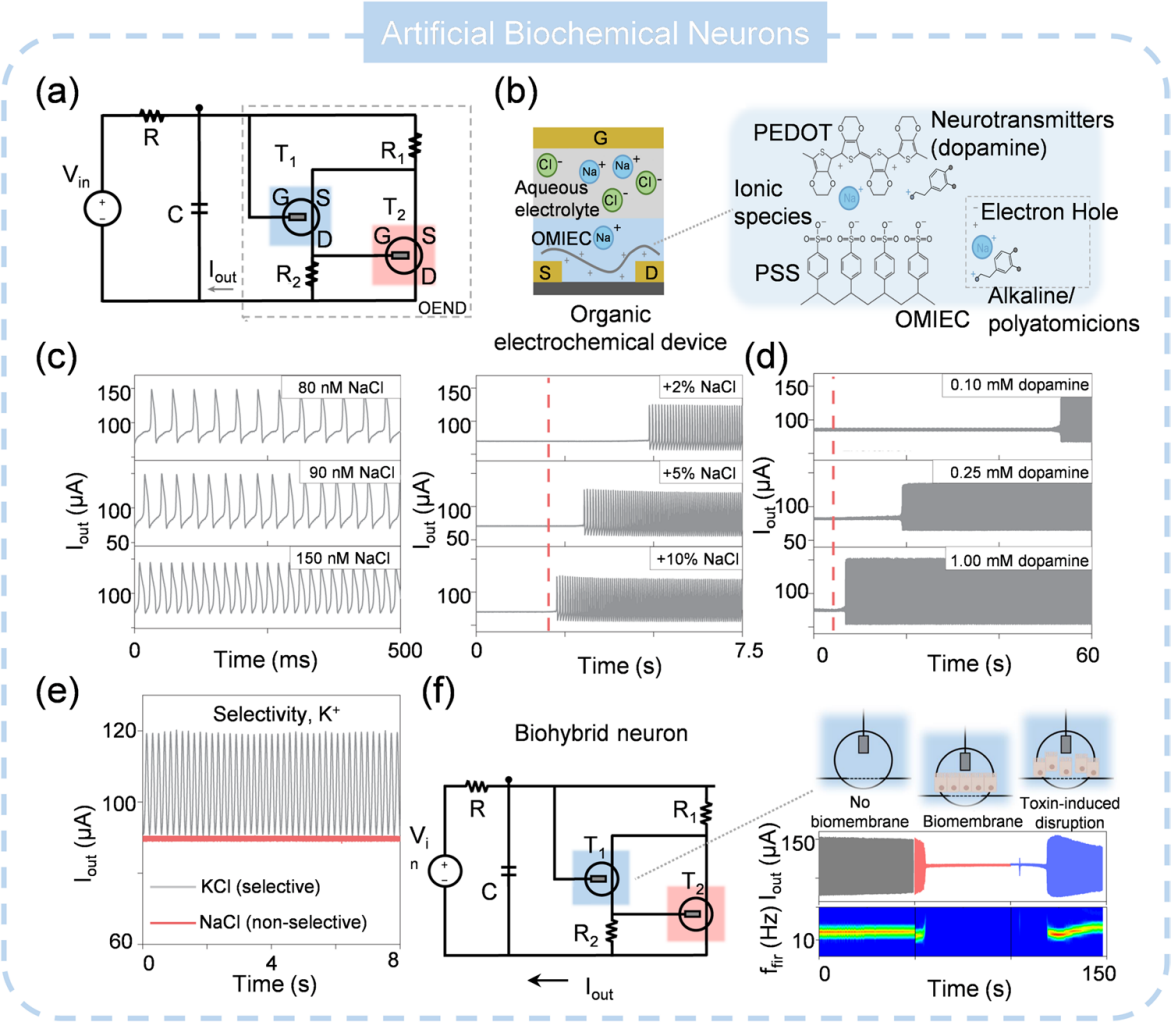
Artificial biochemical neuron. **a** Structure of ABCN. **b** Device and materials used for ABCN. **c** Spiking under different NaCl concentrations. **d** Spiking under different dopamine concentrations. **e** Selectivity of ABCN. **f** ABCN-based biohybrid neuron. **a**–**f** Reproduced with permission from Ref. [205]. Copyright 2022, Springer Nature

Drawing inspiration from the biological receptor, ion channels in the cell membrane are maintained in a closed, equilibrium state to ensure a balanced distribution of cations and anions across the membrane [199, 200]. Upon exposed to external stimuli, these ion channels open, initiating the production of electrical signals that are transmitted to the CNS. Furthermore, the neural activity is influenced by the concentration of neurotransmitters [201, 202]. Despite the development of some ionic sensors, they do not possess the capability to generate spikes [203, 204]. Thus, the development of ABCNs that is sensitive to the concentration of biological species holds significant potential. They could fully replicate the electrophysiological characteristics of biological receptors and work in situ in biological environments. Sarkar and colleagues described an organic artificial neuron (OAN) that functions as an ABCN, exhibiting spiking behavior that is sensitive to ion or biomolecular concentration [205]. The OAN operates in liquid environments and inherently interfaces with biological systems. The device primarily consists of two organic electrochemical transistors (OECTs) connected in a cascade to form an organic electrochemical nonlinear device (OEND), as shown in Fig. 14a. The transistors are made from organic mixed ionic-electronic conductors (OMIECs): poly(3,4-ethylenedioxythiophene) (PEDOT) doped with poly (styrene sulfonate) (PSS), and poly(2-(3,3′-bis(2-(2(2-methoxyethoxy)ethoxy)ethoxy)-[2,2′-bithiophen]-5-yl) thieno [3,2-b] thiophene) (p(g2T-TT)), as presented in Fig. 14b. The OEND’s negative differential resistance allows the OAN to generate stable and repeatable current spiking under voltage bias. These spikes are highly dependent on the NaCl concentration, with the spiking frequency increasing with Na^+^ concentration (Fig. 14c). This is due to the interaction between ions and the PEDOT: PSS, which alters the doping level and drain current of the OECT. The OAN’s behavior is closely similar to that of biological neurons, where changes in ionic concentration gradients between the intracellular and extracellular medium impact neuronal firing threshold and timing. The OAN also responds to biomolecular concentration, with dopamine affecting its spiking frequency (Fig. 14d). A modified OAN with an ionophore-based selective membrane demonstrates K^+^ response selectivity, akin to biological ion channels (Fig. 14e). The OAN’s biointerfacing capability is illustrated by the introduction of a biomembrane, which halts oscillations when inserted between the gate and channel (Fig. 14f). However, there are many obstacles related to organic materials that have not been solved which impede the ABCN to perfectly interact with wet biological surroundings: 1) organic materials generally suffers instability when exposed to environmental factors, i.e., temperature, ultraviolet (UV) radiation, leading to the performance degradation [206, 207]; 2) the heat generation during the operation may disintegrate the structure of organic materials; 3) the organic materials are indeed promising for scalability, but it is challenge for organic materials to achieve high uniformity across large-scale production; 4) integrating organic materials with existing silicon-based technologies can be complex due to differences in processing techniques and material compatibility; 5) organic-based ASN probably possesses higher energy consumption compared to inorganic alternatives (several tens of nanojoules per spike for organic materials versus sub-nanojoules per spike for inorganic materials). Presently, investigations to address these limitations and improve the performance of organic-based ASN is ongoing, aiming to meet the needs of biological applications.

### Artificial Visual Neuron (AVN)

**Fig. 15:**
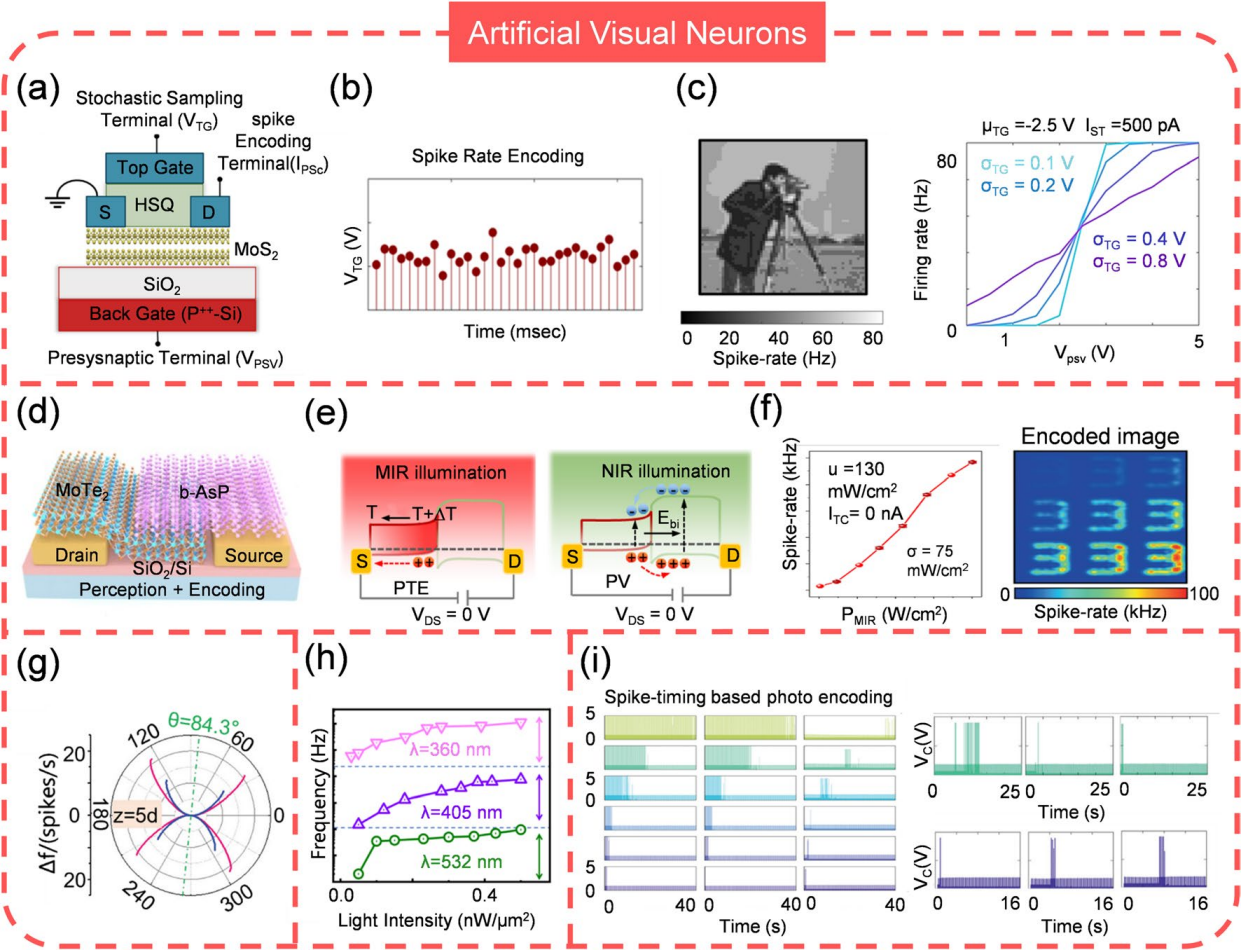
Artificial visual neurons. **a** Structure of MoS_2_ memtransistor based ASN. **b** Spike generation of MoS_2_ memtransistor based ASN. **c** Spike rate encoding and the tunability of spiking behavior. **a**–**c** Reproduced with permission from Ref. [209]. Copyright 2021, Springer Nature. **d** Structure of MIR responsive ASN. **e** Working mechanism and **f** spiking encoding of MIR responsive ASN. **d**–**f** Reproduced with permission from Ref. [211]. Copyright 2023, Springer Nature. **g** Depth perception, **h** color selectivity and **i** spiking timing encoding with ASN. **g** Reproduced with permission from Ref. [216]. Copyright 2022, John Wiley & Sons. **h** Reproduced with permission from Ref. [217]. Copyright 2023, Springer Nature. **i** Reproduced with permission from Ref. [221]. Copyright 2022, John Wiley & Sons

AVNs aim to mimic the way of biological photoreceptors encode visual information, providing a more efficient and energy-efficient way of capturing and interpreting visual data [208]. They are developed for a variety of applications including autonomous vehicles, robotics, surveillance, and medical imaging. Their ability to perform complex visual tasks with minimal power and computational resources makes them particularly attractive for use in resource-constrained environments. Generally, AVN are required to be highly sensitive, responsive, compact, and selective, imitating the biological photoreceptor as far as possible and a lot of efforts have been devoted to achieve this goal. Radhakrishnan et al. [209] reported a AVN using MoS_2_ memtransitor to encode the intensity of white light (Fig. 15a). With the photodiode, the light intensity can be converted into current and consequently impact the spike generation of AVN (Fig. 15b). Such properties allow the AVN to well encode the images with different brightness. Notably, the performance of AVN can be tuned by varying the testing parameters, which shows the potential to meet various demands (Fig. 15c). Lee and colleagues [210] also developed a AVN operating in the visible light spectrum by finely tailoring the electrical characteristics of Ag-doped GeSe_2_ and integrating it with a photodiode. The encoder’s spiking frequency changes when the condition changes from “light off” to “light on”, mirroring the behavior of photoreceptor cells. Simulation data suggests that this system is available for the classification of chest X-ray images, aiding in disease diagnosis. Visible light coding can indeed cover most of the application scenarios, however, for specific situation, invisible light plays a dominate role. For instance, infrared (IR) machine vision, which efficiently interprets, converts, and processes vast amounts of IR optical data about objects, has emerged as a critical technology for making decisions in diverse fields such as autonomous driving, intelligent night vision, military defense, and medical diagnosis. In a pioneering study, Wang et al. [211] have developed an AVN that operates in the mid-infrared (MIR) range with a 2D van der Waals heterostructure (b-AsP/MoTe_2_), as shown in Fig. 15d. The AVN employs a stochastic near-infrared (NIR) sampling terminal to efficiently process the MIR optical information. The b-AsP layer, with its narrow bandgap of ~ 0.15 eV and high MIR optical absorption, acts as the MIR photosensitive layer; while MoTe_2_, with a bandgap of ~ 1.0 eV, serves as the NIR sensitizer. The sensor’s photoresponsivity is a result of the photovoltaic (PV) and photothermoelectric (PTE) effects. The MIR laser-induced temperature gradient, along with the Seebeck coefficients and thermal conductivities, leads to hole diffusion and the generation of a positive PTE photocurrent. NIR illumination generates electron–hole pairs, which are separated by the built-in electric field, resulting in a negative photovoltaic current (Fig. 15e). The spiking frequency is almost linear proportional with light power intensity, which enables the image encoding (Fig. 15f).

Improving the resolution of AVNs can significantly enhance its ability to capture fine details and image clarity. The most straightforward way to increase resolution is to use an array with a higher number of pixels. Such strategy requires the structure of AVNs should be compact. On this account, Zhao and colleagues proposed an AVN based on perovskite oxide NdNiO_3_ (NNO), which has an optical bandgap of 1.7 eV [212]. When exposed to visible light, the NNO memristor generates electron–hole pairs in the NNO layer, resulting in increased conductance, a decrease in threshold voltage, and an improvement on the spiking frequency. This design offers a more compact solution by integrating the photodetector with the memristor, thereby reducing the complexity and power consumption of the circuit. However, NNO poses some drawbacks such as chemical instability, expensive cost, and complex fabricated process. To overcome these issues, Han et al. [142] utilized a polycrystalline Si-based STLFET, which is sensitive to visible light wavelengths and intensities, to produce an AVN. Its intrinsic light responsiveness eliminates the need for external resistors or additional sensors, showcasing high scalability and compactness. Notably, it can generate distinct spike trains for red, green, and blue light with varying intensities but exhibits invariable spikes for IR light across different intensities. To extend the AVN’s capability to broadband wavelengths including IR, researcher substituted poly-Si with InGaAs, which has a narrower bandgap energy of 0.75 eV [213]. This allows the sensor to encode both visible and IR light, even at ultralow intensities, demonstrating high light sensitivity. This AVN operates near 1 V, offering low energy consumption and high speed compared to previous poly-Si AVNs.

As mentioned previously, AVNs tend to reproduce the biological processes of the photoreceptors, particularly in terms of sensory perception and information encoding. Therefore, an ANV that is biologically plausible is more likely to accurately capture the essence of biological sensory mechanisms. Depth perception in human eyes enables the estimation of relative distances in three-dimensional space and the judgment of an object’s distance from the viewer [214]. This capability is essential for navigation, object interaction, and spatial positioning. The primary cue for depth perception is binocular disparity, where the brain calculates depth by comparing slightly different images from each eye due to their horizontal separation [215]. Recently, Chen et al. [216] reported an AVN that resembles such visual depth perception, with its spiking rate dependent on the object’s distance (Fig. 15g). Comparing the spiking rates from the “two eyes” permits the calculation of depth information. Additionally, asthenopia, a condition of discomfort, pain, or tiredness in the eyes after prolonged focus, is also shown. The spiking rate slows down in the fatigue state, which reduces sensitivity and causes defocus. The same group developed another AVN attempts to imitate the color selectivity of human eyes, which is a result of the complex interaction between light, cornea, lens, and cones [217]. It is able to differentiate various light wavelengths and intensities with no overlap in spike frequency, effectively conveying the color and intensity of light stimulation (Fig. 15h). This capability is particularly useful for recognizing mixed-color patterns, where distinct wavelengths should be clearly distinguished. Moreover, it has an ultra-low power consumption of less than 400 picowatts per spike in visible light and operates at a spiking frequency ranging from 0.1 to 1200 Hz, which is comparable to biological cones. This AVN could serve as a fundamental component for hardware that implements sophisticated color perception within spiking neural networks (SNNs).

Spiking-timing-based encoding is a critical feature of visual perception. Such characteristics can be exemplified by the face recognition of rhesus monkeys [218]. Though there are more than ten synaptic steps to convert the visual information from photoreceptors to cortex, they can implement the face recognition within 160 ms, which reveals that each step must accomplish the processing in 10 ms. Since cortical neurons fire at rates of 0 to 100 spikes per second, rate-spiking encoding alone does not fully account for this behavior. It has been proved that spiking-timing encoding is more useful than spiking-rating for fast response [219, 220]. Building on this insight, Radhakrishnan et al. [221] developed a photoencoder comprising two cascaded three-stage inverters and an XOR logic gate with a total of 21 memtransistors based on photosensitive 2D monolayer MoS_2_. Experimental results indicate that the time of the first spike is inversely proportional to light intensity, which suggests that light intensity can be encoded by the timing of the first spike (Fig. 15i). Furthermore, the spiking performance can be adjusted by tuning the applied voltage, demonstrating adaptive photoencoding capability in both scotopic and photopic conditions. This is akin to visual adaptation in biological systems and is essential for maintaining clear vision across a range of environments, from bright sunlight to dim moonlight.

### Artificial Multimodal Neuron (AMN)

**Fig. 16:**
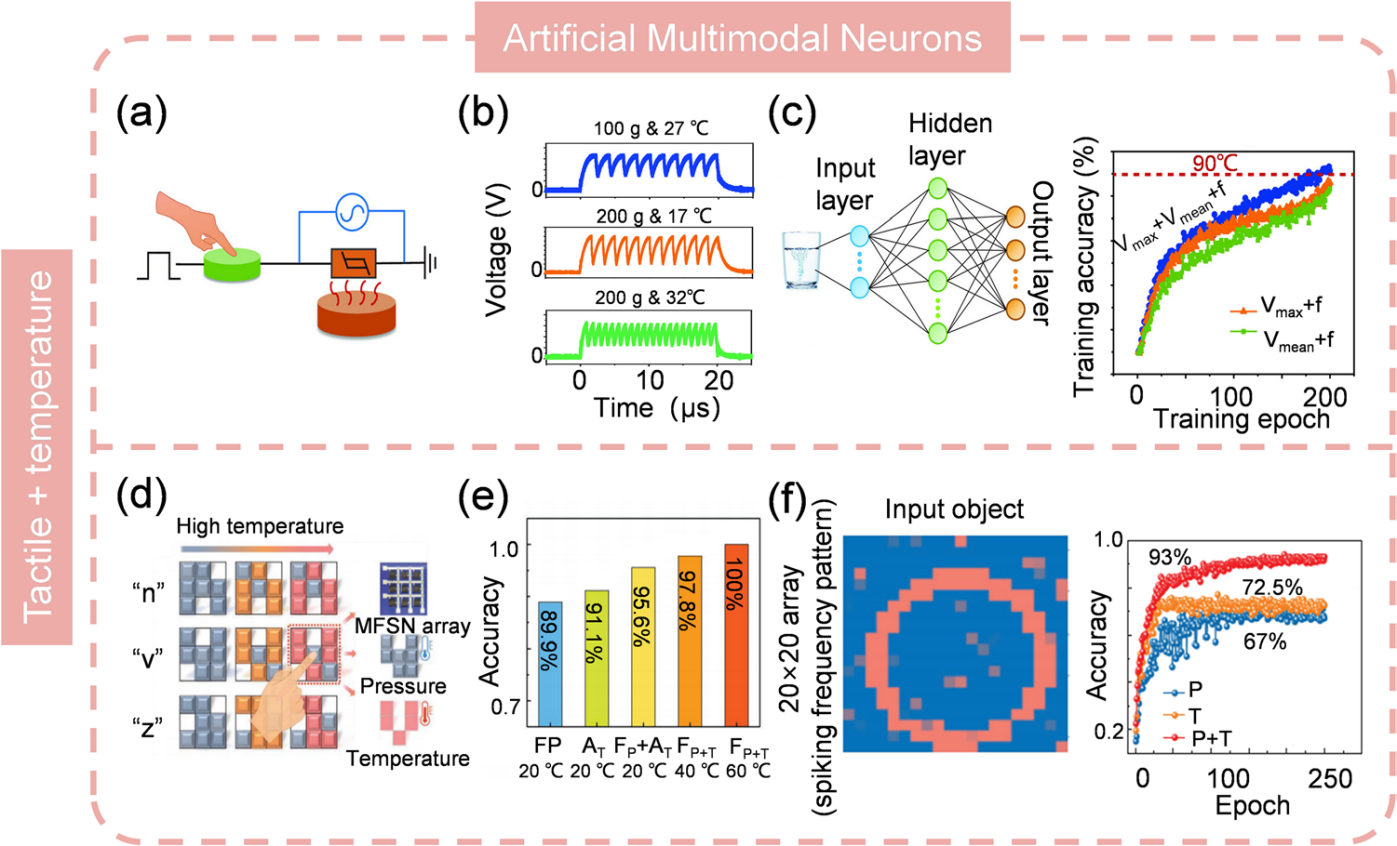
Artificial multimodal neurons for tactile and temperature sensing. **a** Structure of AMN. **b** Spiking encoding of temperature and tactile information with AMN. **c** Neural network for object classification. **a**–**c** Reproduced with permission from Ref. [226]. Copyright 2022, John Wiley & Sons. **d** 3 × 3 AMN array. **e** Temperature-enhanced recognition accuracy with AMN. **f** Classification simulation with 20 × 20 AMN array. **d**–**f** Reproduced with permission from Ref. [227]. Copyright 2022, John Wiley & Sons

Single-mode perception offers clues from a specific aspect of the environment or object but often overlooks other critical details, potentially leading to misinterpretations [222, 223]. Multimodal perception, the ability to interpret and integrate information from multiple sensory modalities such as sight, sound, touch, taste, and smell, can effectively mitigate the issues arising from single-mode perception [224, 225]. It provides a more comprehensive sensing of the environment, enhancing decision-making and response capabilities. For robots and autonomous vehicles, multimodal perception is essential for achieving human-like intelligence and versatility. Currently, AMNs that can concurrently perceive and convert various stimuli into spikes have also been presented. For example, Duan and colleagues developed an AMN, which integrates a piezoresistive sensor and a VO_2_ volatile memristor, to fulfill haptic-temperature fused sensation (Fig. 16a) [226]. It takes advantage of the voltage dividing effect and the inherent thermal sensitivity of VO_2_ to detect and encode pressure and heat from an object at the same time. Different weight and temperature of cups generates various output spiking waveforms (Fig. 16b), which are labelled as inputs to feed into the multilayer perceptron (MLP) for processing and classification. Since the AMN integrates two modalities, the spiking frequency alone is insufficient for accurate classification. Hence, the spiking amplitude should also be considered as a classification criterion (Fig. 16c). In addition, adding more parameters in the input data leads to better training performance. An analogous work was also reported by Zhu et al. [227] but with an array demonstration, enhancing its suitability for intelligent applications. With a 3 × 3 AMN array, they were able to clearly distinguish “n”-, “v”-, and “z”-shaped objects with noise at various temperatures by leveraging the frequency and amplitude attributes of the fused output spikes (Fig. 16d). Two 3 × 3 matrices corresponding to single modalities (pressure or temperature) were also constructed for accuracy comparison. The results indicate that the multimodal fusion approach outperforms unimodal methods in recognition rate, indicating a higher level of intelligence. Intriguingly, increasing temperature is beneficial for recognition accuracy. The AMN array exhibits a thermally assisted frequency enhancement, with the output frequency from noiseless pixels increasing with temperature when the pressure remains constant (Fig. 16e). Such frequency difference between noiseless and noisy pixels becomes more significant because heating effectively suppresses the frequency signals from the noisy pixels when heated. A 20 × 20 array simulation was conducted based on the experimental data with a SNN classifier (Fig. 16f). The system achieved a higher recognition rate (93%) for distinguishing cup features using patterns with multiple modes compared to adopting only the temperature mode (72.5%) or the pressure mode (67%).

**Fig. 17:**
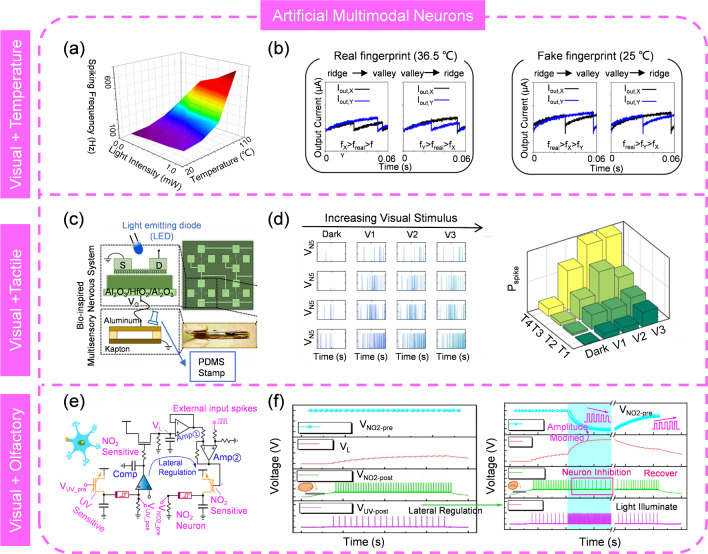
AMNs for visual-thermal, visual-tactile and visual-olfactory sensing. **a** Distribution of spike frequency under different temperatures and light intensities. **b** Fingerprint recognition with visual-thermal AMN. **a**–**b** Reproduced with permission from Ref. [228]. Copyright 2023, American Chemical Society. **c** Structure of visual-tactile AMN. **d** Spiking performance of visual-tactile AMN. **c**–**d** Reproduced with permission from Ref. [233]. Copyright 2023, Springer Nature. **e** Configuration of visual-olfactory AMN. **f** Spiking behavior under stimulation of light and gas. **e**–**f** Reproduced with permission from Ref. [234]. Copyright 2023, American Chemical Society

Visual-thermal cross-modal perception, which integrates visual and thermal data, allows for the detection of objects and living beings that may be obscured or indistinguishable in the visual spectrum. It can enhance human–machine interaction by endowing machines with a human-like perception, leading to more intuitive and natural interactions. For future intelligent system with high energy efficiency, an artificial visual-thermal neuron is pivotal. Motivated by this, an AMN to encode the external light and temperature via a STLFET has been presented by Han and colleagues [228]. The movement of electrons within the transistor can be precisely controlled by light and temperature stimuli, allowing for the adjustment of spiking frequency. An increase in temperature or light intensity leads to a higher spiking frequency, but temperature having a more significant impact than light (Fig. 17a). Such AMN holds promise for fingerprint recognition, where valleys and ridges are represented by bright and dark light, and the authenticity of fingerprints is determined by temperature differences (Fig. 17b). This is the first attempt of hybrid visual-thermal encoding with a single device, although challenges related to stability, uniformity, and large-scale integration remain to be overcome. Apart from tactile-thermal and visual-thermal perception, visuo-tactile integration is another category of multi-modal perception, which emulate the procedure of brain to process information from eyes and skins together [229, 230]. It is particularly important in conditions where visual or tactile information alone might be insufficient to achieve a thorough insight. For example, when grasping an object, visual information about the object’s shape and size is combined with tactile information about its texture to create a more accurate discernment of the object [231, 232]. Hitherto, researchers are developing artificial visuo-tactile neurons to enable machines to perform tasks that require the same level of visuo-tactile integration as humans. As an illustration, Sadaf and colleagues proposed a system that converts visuo-tactile information into spikes using a photosensitive MoS_2_ memtransistor and a triboelectric tactile sensor (Fig. 17c) [233]. The system captures the features of multimodal sensory integration, demonstrating super-additive response, inverse effectiveness, and temporal congruency. Increasing the visual stimulus or tactile stimulus could significantly enhance the spike probability, and the synergic effect of visual and tactile stimulus yields a higher spike probability than that of either stimulus alone (Fig. 17d). The visuo-tactile encoder circuit comprises four memtransistors, occupying a considerable chip area and presenting a complex design. Furthermore, it’s possible to obtain the same spike probability even if under different visuo-tactile conditions, making it challenging to differentiate solely based on spike probability. To avoid confusion, additional clues like the time-to-first spike should be entailed. Another concern is the encoding time, which can take dozens to hundreds of seconds, severely lowering the processing speed. Therefore, accelerating the encoding process through device optimization is crucial for practical applications.

Merging the gas and light signals by AMN is also demonstrated by Yuan et al. [234] with Mott memristor and an amorphous indium gallium zinc oxide (IGZO) thin-film transistor (TFT) (Fig. 17e). The IGZO TFT shows responsiveness to both NO_2_ gas and UV light. Under NO_2_ gas stimulation, the AMN exhibits inhibitory behavior, whereas it demonstrates excitatory behavior upon UV light. This is different to other works, where all modalities typically induce excitation. By coupling the NO_2_ gas spike encoder and the UV light spike encoder, both gas and light stimuli can be encoded into spikes, and their relationship is interactive through lateral regulation (Fig. 17f). They found that an increase in spike frequency for UV light leads to a decrease in spike frequency for NO_2_ gas. The integration of light-gas perception is achieved through circuit interconnection, delivering an alternative approach to address the spike coupling issue by separating two paths. However, this scheme sacrifices hardware area costs and does not really achieve a true fusion of perceptions.

## Key Metrics of ASNs

### Power Consumption

Power consumption is a primary concern in electronic devices due to its direct impact on heat dissipation, performance degradation, and overall energy efficiency. To calculate the total power consumption of ASNs, one must consider the power demands of both the sensors (if applicable) and the neuronal devices. The power consumption of sensors is typically evaluated based on their operating voltage and current, but for ASNs, these parameters are often not provided, making power estimation challenging. Accordingly, here, we focus on evaluating the power consumption of neuronal devices using spikes. The average energy per spike is calculated as the V × I × t/N, where V is input voltage, I is output current, t is stimulation time, N is spike number [235, 236]. Achieving ultra-low power consumption for energy efficient sensing and encoding is the ambition, since it can offer a longer operation time under limited power supply and mitigate the thermal effect to increase the service life for practical applications. In order to lower the power consumption of artificial neuronal devices, decreasing the operation current is an effective way and several methods such as nanodots involvement, element doping, multi-layered oxide design, and incorporation of diffusion layer have been demonstrated [237–239]. As a part of the ASN, improving the energy efficiency of sensors is also important and the main approach to achieve this is through materials engineering [240–242]. For example, Guo et al. [242] fabricated a pressure sensor with 10 nW via MXene nanosheets and Luo et al. [240] reported a low-power (3 nW) piezoresistive sensor using carbon-decorated fabric. A more promising manner is to integrate sensing and encoding functions within a single device, namely, in-sensor encoding. This idea has been implemented in the visual domain, whereas its application to other sensory modalities is still under investigation [142]. The main challenge lies in the distinct material candidates for sensors and neuromorphic devices.

### Sensitivity and Dynamic Range

The sensitivity of an ASN is a measure of its ability to detect and respond to a specific stimulus, quantifying the variation in output spiking frequency for a given change in the input [243–245]. It is expressed as Δ*f*/Δ*x*, where Δ*f* represents the change of output spiking frequency, and Δ*x* denotes the change in the input. Sensitivity is crucial for ASN design as it determines the dynamic range and the minimum level that can be perceived. An ideal ASN is supposed to achieve a high sensitivity within a wide dynamic range. However, there is a tradeoff between high sensitivity and dynamics range [246]. The sensitivity of ASNs with wide dynamic range is lower, yet the dynamic range of those with high sensitivity is narrow. Fortunately, the development of novel materials probably provides an encouraging solution to overcome this challenge. Recently, a flexible ferroelectric pressure sensor with ultrahigh sensitivity over a broad range was reported [247]. Such a goal can also be realized with porous conductive hybrid composite [248]. It can be envisioned that ASNs with wide dynamic range and high sensitivity are accessible with the advancement of material science. The requirements for sensitivity and dynamic range of ASNs vary with the targeting applications. A sensor with high sensitivity can detect minor changes in the input stimulus, making it suitable for applications that demand high precision and sensitivity. On the other hand, a sensor with low sensitivity is more robust to noise and less sensitive to tiny changes, which is potential for applications that require robustness and a wide input range. Therefore, in practical applications, the required sensitivity of ASNs needs to be considered in multiple dimensions such as detection limit, signal-to-noise ratio, robustness, and dynamic range to ensure their reliability and effectiveness in specific environments.

### Linearity

The linearity of ASNs is a critical parameter of its performance, referring to the capacity to produce an output spike frequency that is linearly proportional to a changing input. Linearity estimation, which is also expressed as the percentage of nonlinearity, involves comparing the ASNs measured output to its expected output across a range of inputs and determining the closeness of the data to a best-fit line [249]. The linearity of ASNs can be measured by D_out(max)_/ Out_f.s_ × 100%, where D_out(max)_ is the maximum output deviation of spikes, and Out_f.s_ is the full-scale output of spikes [250]. This calculation allows for the quantification of linearity error, which is the discrepancy between the ASNs’ actual output and theoretical output. It is noteworthy that high linearity is essential for applications that demand accurate and repeatable measurements, as it ensures that the response faithfully represents the input’s true value, thus maintaining the authenticity of the data collected. A nonlinear response can lead to measurement errors, particularly in critical applications where precise and predictable performance is essential such as control systems and scientific research, necessitating calibration or the use of linearization techniques to ensure reliable and accurate data interpretation. The linear response of ASNs is jointly determined by the performance of artificial neuronal devices and sensors. Currently, a number of strategies, for instance, microstructure engineering, and materials design, have been proposed to improve the linearity of sensors [247, 251–253]. Nonetheless, there is no theoretical or experimental demonstration to improve the linear spiking output of artificial neuronal devices. How to optimize the linear response of ASNs is still challenging.

### Response Time

The response time of ASNs determines how quickly it can detect and react to input stimuli. It is defined as the time taken for the ASNs to output spikes that accurately reflect the input, which is vital in applications that need rapid response such as in real-time monitoring systems. A fast response time ensures that the ASNs can quickly capture and relay information about the stimuli, enabling timely decision-making and ensuring the reliability of the system. Whereas a slow response time may be sufficient for less time-sensitive applications like long-term data collection but can lead to delayed reactions and potential errors in critical situations. Given that there are two approaches for spike generation (oscillation and LIF) of ASNs, the response time of oscillatory ASNs (~ μs) is much faster than that of LIF ASNs (~ ms) because the integration process of LIF is time-consuming. Here, we neglect the response time of the sensor. Actually, the response time of the sensor ranges from several milliseconds to seconds. For pressure sensors, it can be optimized by microengineering through making the material more compressible and elastic [254]. As discussed above, it is easy to find that the response time of ASNs is governed by the performance of sensors. How to further improve the characteristics of sensors is an urgent issue needs to be solved for ASNs.

### Resolution

The resolution of a ASN refers to the smallest change in the physical quantity being measured that the ASNs can detect and encode. It is a measure of the ASNs’ ability to distinguish two different conditions. High-resolution ASNs can detect and encode very small changes in the dynamic range, providing more precise and detailed data. Conversely, ASNs with lower resolution may only be able to detect larger changes, leading to less granular and potentially less accurate measurements. Resolution is often expressed in terms of the smallest increment the ASNs can measure, such as centigrade for ATMNs or Pascal for ATTNs. For scenarios where detailed and precise measurement is required such as medical diagnostics, and security surveillance, a high-resolution ASN is preferable. Whereas in certain applications where broad trends or approximate values are acceptable, low-resolution ASNs is adequate. It should be pointed out that higher resolution will inevitably increase the data volume and put forward higher requirements for processing. Thus, the resolution choice of ASNs should balance the practical constraints like processing power, storage, and application needs. The resolution of ASNs is highly dependent on the resolution of sensors, therefore improving the resolution of sensors is beneficial for the ASNs. It is reported that the resolution of sensors can be enhanced by the manufacturing process or microstructure design [255, 256]. These schemes are believed to be applicable to the resolution improvement of ASNs.

### Stability

The stability of ASNs represents their ability to maintain a consistent spiking output during their operational life, resisting to the aging and variation of environmental conditions such as humidity, temperature, mechanical stress. Obviously, a high stability of ASNs is required for real applications because it ensures that the output of ASNs suffers minimal drift or change in various surroundings. Instability of ASNs can lead to measurement error, safety issues, and increased operational costs due to the need for more frequent recalibrations or replacements. However, for applications where a short lifetime is demanded, e.g. implantable electronics to avoid chronic risk, ASNs that can be dissolved in physiological solution are desired. Considering the robustness of commercial sensors, the stability of ASN can be evaluated by estimating the performance of artificial neuronal devices. Though a thermal stable STL MOSFET-based artificial neuron has been reported, most of the current artificial neuronal devices are susceptible to temperature fluctuations because the threshold switching process is thermal-sensitive. A summary of the performance metrics of various ASNs is shown in Table 2.

**Table 2:** Summary of performance metrics of various ASNs

Type	Sensor configuration	Power consumption	Sensitivity	Dynamic range	Linearity (%)	Response time	Year	References
Tactile	Mott memristor + Pressure sensor	0.14 mJ	3 kHz/kPa	3.8 ~ 10.8 kPa	13.8	0.0321 ms	2022	[171]
	Mott memristor + Pressure sensor	0.6 nJ	23.3 MHz/kPa	0.3889 ~ 0.700 kPa	25.17	1.4 us	2021	[160]
	Mott memristor + TENG	4 nJ	–	–	–	21 ms	2020	[159]
	OTS memristor + Pressure sensor	3.54 nJ	70.6 kHz/kPa	6 ~ 20 kPa	20.8	< 1 us	2022	[257]
	Mott memristor + Pressure sensor	28.5 nJ	60.8 kHz/kPa	4.8 ~ 6.4 kPa	17.3	4.71 us	2022	[164]
	Mott memristor + Pressure sensor	2.1 nJ	3 kHz/N	1.37 ~ 2.85 N	12.5	1.5 us	2024	[161]
	Diffusive memristor + Pressure sensor	30 mJ	–	–	–	–	2020	[159]
	Mott memristor + Pressure sensor	40 nJ	26.6 kHz/mN	0.25 ~ 1.02 mN	12.5	–	2022	[258]
	STL MOSFET + TENG	0.98 nJ	1.6 kHz/kPa	3.2 ~ 5.09 kPa	25.41	0.107 s	2022	[172]
	Diffusive memristor + TENG	0.84 mJ	–	10 ~ 40 kPa	–	3.72 s	2022	[165]
Auditory	STL MOSFET + TENG	67.5 nJ	680 Hz/dB	70 ~ 110 dB	27.22	0.037 s	2023	[187]
Gustatory	STL transistor + Biosensor	40 pJ	133 Hz/pH	pH: 3 ~ 9	pH: 63.29	pH: 0.001 s	2022	[191]
			100 Hz/log[Na^+^]	Na^+^: 10^–4^ ~ 10^–1^ Mol	Na^+^:22			
						Na^+^:0.001 s		
Biochemical	Organic Electrochemical Transistors	750 nJ	0.38 Hz/mM NaCl	80–160 mM NaCl	29.6	2 s	2022	[205]
Olfactory	Diffusive memristor + Gas sensor	0.6 nJ	–	–	–	200 s	2022	[197]
	STL MOSFET + Gas sensor	6 uJ	31.25 Hz/ppm @NH_3_	0–2 ppm @NH_3_	10 @NH_3_	0.007 s	2022	[198]
					20 @CO 6 @Acetone			
			7.5 Hz/ppm @CO	0–20 ppm @CO				
			583 Hz/ppm @Acttone		21 @NO_2_			
				0–3 ppm @Acttone				
			50 Hz/ppm @NO_2_					
				0–2 ppm @NO_2_				
Thermal	Mott memristor	40 nJ	–	313 ~ 393 K	–	13.5 ms	2023	[182]
	STL MOSFET	5 nJ	17.21 Hz/°C	30 ~ 110 °C	18.3	0.1 ms	2021	[178]
	Diffusive memristor	90 pJ	1.73 Hz/°C	20 ~ 80 °C	24	3.2 ms	2022	[180]
	Mott memristor	3 nJ	0.23 kHz/°C	5 ~ 40 °C	3.6	0.001 ms	2022	[181]
	Diffusive memristor	–	–	35 ~ 65 °C	–	0.5 s	2022	[179]
Visual	STL MOSFET	0.27 nJ	1.6 Hz/μW @1550 nm	397 ~ 1550 nm	–	0.005 s	2021	[213]
				0.5 -20 mW @1550				
	Diffusive memristor	1.7 uJ	10.83 Hz/cm	0 ~ 30 cm	19.38	0.1 s	2022	[216]
	2D memtransistor	100 nJ		0–25 W/m^2^		0.8 s	2022	[221]
	2D memtransistor	–	1.12 kHz/W/cm^2^	0 ~ 80 W/cm^2^	10.8	10 us	2023	[211]
	STL MOSFET	10 nJ	18.4 Hz/mW	0 ~ 1.24 mW	10.6	0.019 s	2020	[142]
	Diffusive memristor	1 mJ	15 Hz/mW	0 ~ 2.5 mW	23.07	0.11 s	2021	[259]
	Mott memristor	130 nJ	–	0 ~ 100 mW	–	0.044 s	2023	[212]
	Diffusive memristor	10 nJ	–	–	–	–	2022	[210]
	Diffusive memristor	0.6 pJ	2 kHz/ nW/μm^2^ @360 nm	360–532 nm	22.05 @360 nm	0.25 ms	2023	[217]
				0.03 ~ 0.5 nW/μm^2^				
			22.2 Hz/nW/cm^2^@405 nm		17 @405 nm			
			0.22 Hz/ nW/μm^2^ @532 nm		40 @532 nm			

## Potential Applications of ASNs

### Machine Learning

**Fig. 18:**
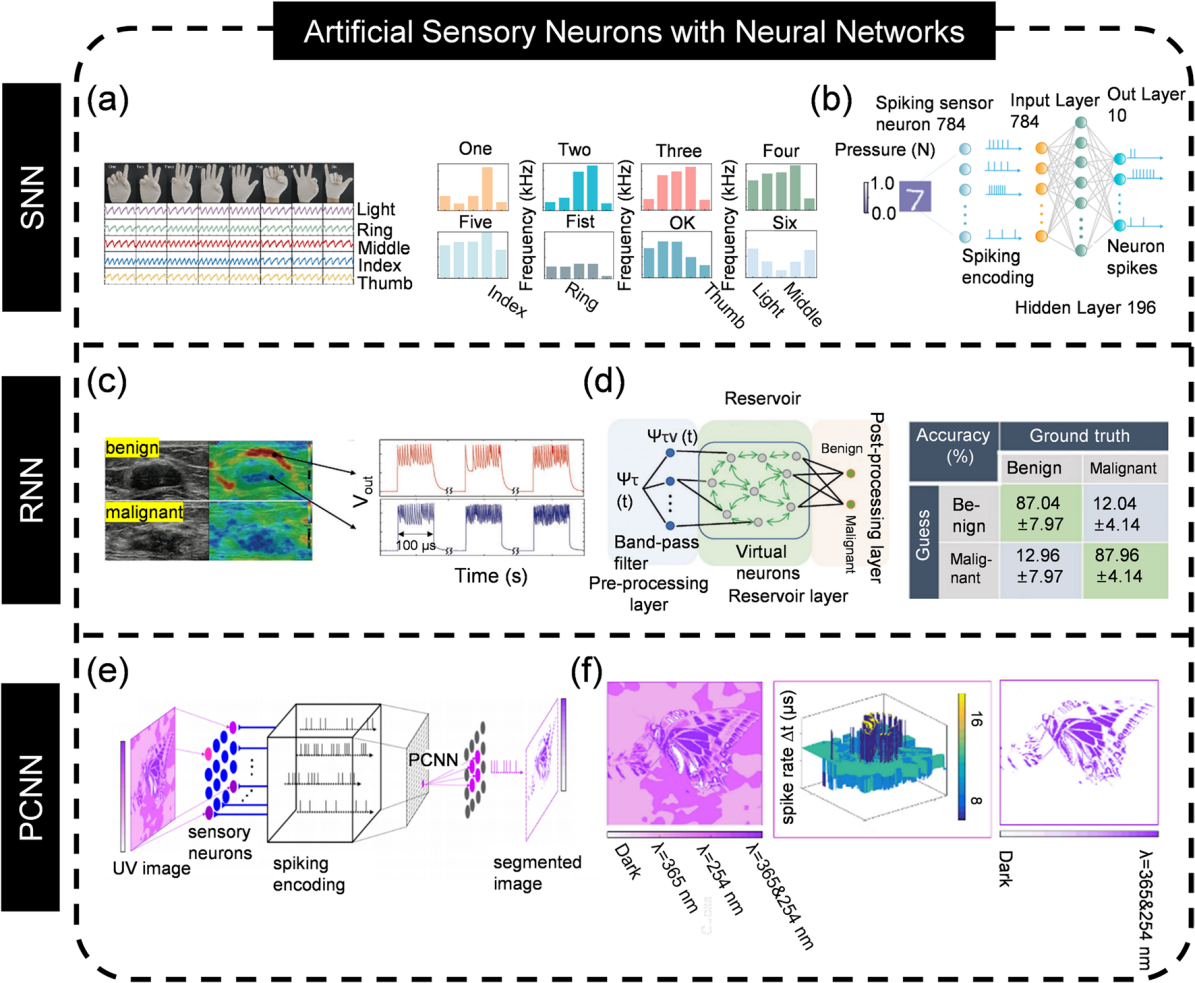
ASNs with neural network. **a** Encoding of finger curve. **b** Classification by SNN. **a**–**b** Reproduced with permission from Ref. [267]. Copyright 2022, Springer Nature. **c** Tumor stiffness encoding with ASN. **d** Tumor classification by reservoir computing. **c**–**d** Reproduced with permission from Ref. [257]. Copyright 2022, John Wiley & Sons. **e** Working flow of image segment. **f** Implementing image segment by ASN and PCNN. **e**–**f** Reproduced with permission from Ref. [274]. Copyright 2020, American Chemical Society

Combining machine learning with sensors has been used to interpret the sensing data [260–262]. SNNs are ANN models that are inspired from the functionality of biological neural network [263, 264]. Unlike conventional ANNs, which use continuous signals, SNNs process information through discrete spike signals, which is the same as the communicated way between biological neurons by action potentials. SNNs excel at handling temporal information and event-related dynamics, which has garnered considerable interest in the fields of cognitive science and neuroscience [265, 266]. Since ASNs can convert external data into spikes, they are well-suited for integration with SNNs to implement energy-efficient processing systems. For instance, Yuan et al. [267] developed a calibratable ASN for gesture recognition. It can detect curvature and convert it into spike signals, with higher curvature resulting in a lower spiking frequency (Fig. 18a). When attached to human fingers, these ASNs enable the detection of different hand gestures by analyzing the spiking patterns. Statistical analysis of the spiking frequency across various gestures reveals distinct patterns, demonstrating the effectiveness of the spike-based neuromorphic perception system for gesture recognition. These spikes can then be input into an SNN for classification after the SNN has been trained using backpropagation algorithms based on the spiking behavior of the ASN (Fig. 18b).

Reservoir computing, drawing inspiration from the human brain’s adaptive learning capabilities, is a burgeoning field in computational neuroscience and machine learning that employs recurrent neural networks (RNNs) [268, 269]. This approach has three primary components: a reservoir, an input layer, and an output layer. The reservoir, as the core of the system, comprises neurons with recurrent connections that can process and store temporal data. The input layer receives external signals and delivers them to the reservoir, while the output layer, which could be a readout layer or a simple linear transformation, generates the final output based on the reservoir’s state. The reservoir’s non-linear dynamics allows it to execute intricate computations and learn from data through a procedure known as reservoir computing training [270, 271]. In this context, spikes can serve as the input signals to train the reservoir network, which can be acquired by ASNs. Lee et al. [257] developed a ATTN capable of identifying and classifying tumors. The sensor’s output spiking frequency correlates with the elastic stiffness of materials, making it suitable for recognizing the modulus distribution patterns of biological objects with various disease states (Fig. 18c). The color-coded ultrasound elastography images indicate the stiffness of the imaged materials, and the shape of this stiffness distribution is commonly used to diagnose the malignancy of breast tumors. With the ATTN, such stiffness distribution can be translated into a spike distribution. During the processing, a data augmentation technique and temporal sequence extraction are employed to preprocess the images, which are then mapped to virtual neurons within the reservoir for classification (Fig. 18d). These findings prove the huge potential of ASNs and reservoir computing in disease diagnosis.

Pulse-Coupled Neural Networks (PCNNs) are neural network models that simulate the neural activity in the visual cortex of mammals [272, 273]. In PCNNs, neurons communicate through pulses or spikes, each neuron responding to specific input stimuli. The key concept in PCNNs is the synchronization of neuron activity, where neighboring neurons synchronize their firing patterns based on the timing of incoming pulses. This synchronization enables PCNNs to identify patterns and features in data by analyzing spatial and temporal relationships. The network’s capability to capture both spatial and temporal information makes PCNNs suitable for complex pattern recognition and analysis. Evidently, ASNs, which can generate spikes, can serve as an input for PCNNs. For example, Wu et al. [274] demonstrated a AVN for image segment with a Mott memristor and IGZO_4_ photosensor (Fig. 18e). The AVN is highly sensitive and can respond to UV intensities as low as 0.2 mW cm^−2^. Different UV wavelength combinations (254 nm or 365 nm or both) elicit varying spike frequencies, suggesting the AVN’s potential as an image detector. Accordingly, a butterfly image (600 × 600 pixels) with mixed UV light was reconstructed and encoded, and then segmented by PCNNs (Fig. 18f). The butterfly was successfully extracted from the UV image, even with a noise background. Combining ASNs with neural network offers several advantages such as energy efficiency, robustness to noise, event-driven processing, but they also face challenges, e.g. traditional von Neumann architectures are not well-suited for spikes-based processing, necessitating the development of new types of processors, such as neuromorphic chips, which are still in the early stages of development.

### Nociceptive Sensation

**Fig. 19:**
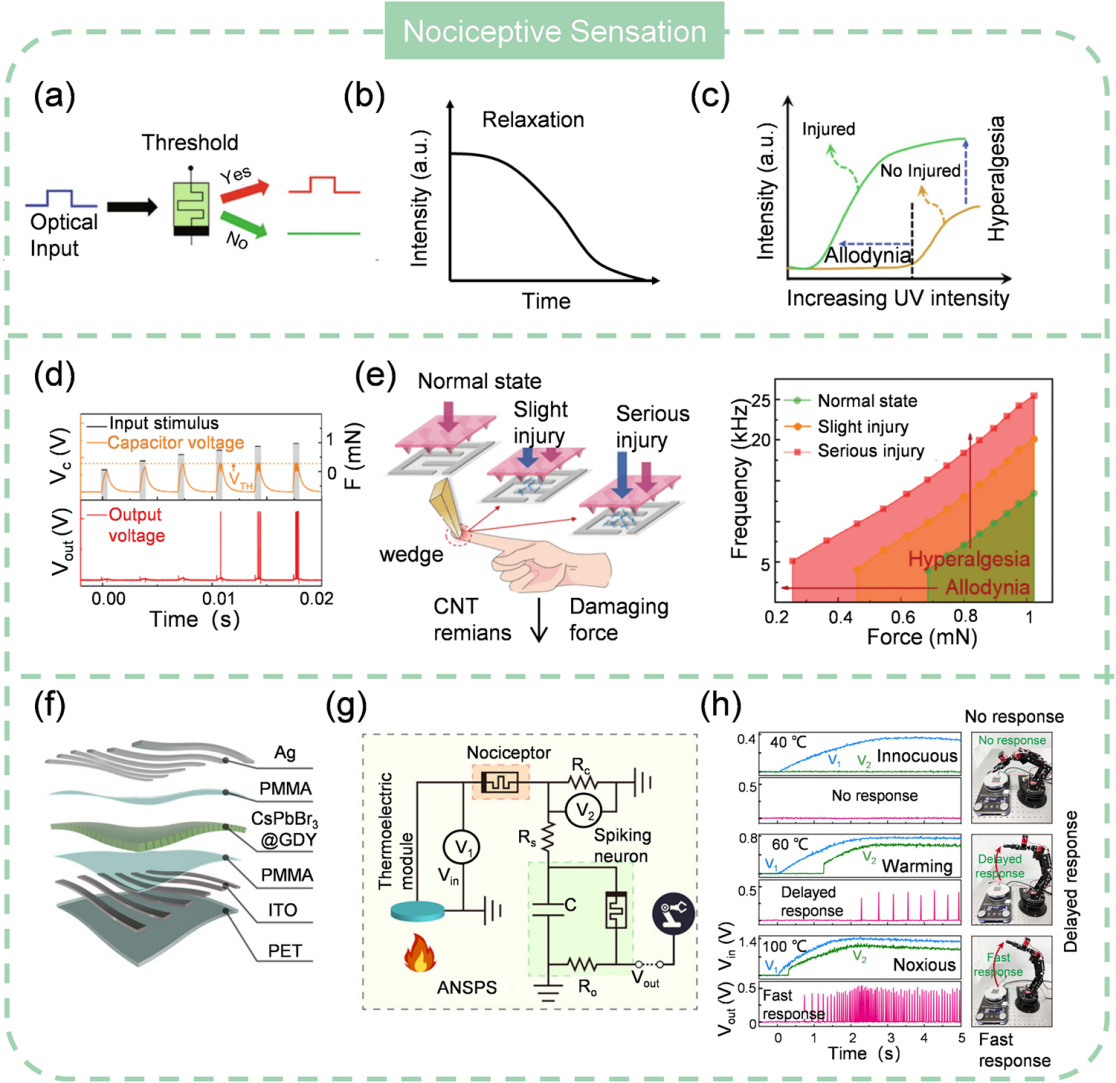
Nociceptive system with ASNs. **a** Threshold property of nociceptor. **b** Relaxation of nociceptor. **c** Nociceptive sensation. **d** Threshold emulation with ASN. **e** Schematic of injury state and simulation of nociceptive sensation. **d**–**e** Reproduced with permission from Ref. [258]. Copyright 2022, IEEE. **f** Structure of memristor. **g** Circuit of ASN-based nociceptive system. **h** Nociceptive response of robot arm. **f**–**h** Reproduced with permission from Ref. [281]. Copyright 2022, John Wiley & Sons

Nociceptive sensation refers to the perception of pain triggered by harmful stimuli, acting as a vital mechanism that alerts the body to potential damage or injury [275, 276]. It results from the activation of specialized nerve fibers known as nociceptors, which are sensitive to various stimuli like extreme temperature, pressure, light, or chemical irritants [277, 278]. When stimulated, nociceptors convert the signals into spikes and send them to the brain for processing and interpretation. There are four typical features of nociceptors: “threshold,” “relaxation,” “no adaption,” and “sensation” [279, 280]. The threshold of a nociceptor indicates the level of stimulation needed to activate the nociceptive nerve fiber and elicit a pain response (Fig. 19a). Unlike other sensory receptors with lower activation thresholds, nociceptors typically have higher thresholds, responding only to intense or potentially damaging stimuli. Once the stimuli are removed, the activity or sensitivity of nociception decreases, a process known as relaxation (Fig. 19b). This relaxation is a protective mechanism that helps maintain an appropriate pain response, preventing excessive suffering or disability. The no adaption process describes the situation where nociceptors do not decrease their activity or sensitivity over time in response to a sustained pain stimulus, which plays a significant role in the development and maintenance of chronic pain. Nociceptive sensation also encompasses allodynia and hyperalgesia (Fig. 19c). Allodynia refers to the phenomenon where non-painful stimuli can evoke pain under certain conditions, while hyperalgesia is the exaggerated pain response to normally painful stimuli.

The use of ASNs to emulate the characteristics of nociceptors has been well presented. As an illustration, Zhu et al. [258] reported an artificial spiking nociceptor using a pyramidal pressure sensor and a Mott memristor. The nociceptor only generates spikes when the applied pressure exceeds a critical value, mimicking the “threshold” feature of biological nociceptors (Fig. 19d). Furthermore, the “relaxation” and “no adaption” properties were also replicated, as the spiking activity ceased upon the removal of pressure and remained constant over long-term pressure. To simulate the nociceptive sensation, a wedge is used to apply a significant force to the sensor, causing localized damage and creating an “injury” state (Fig. 19e). In this case, applying the same force to the pressure sensor, the severity of the injury is directly proportional to the output frequency of the nociceptor, reflecting the “hyperalgesia” characteristic. Compared to the normal state, the injured nociceptor exhibits a lower pressure threshold for spike generation, demonstrating the “allodynia” characteristic. Likewise, Wang et al. [281] proposed a thermally nociceptive system based on ATMN in which a core–shell CsPbBr_3_@graphdiyne nanocrystals is used (Fig. 19f). An integrated thermoelectric module is used to detect external thermal stimuli and convert them into voltage signals, which can be used to activate the ATMN (Fig. 19g). The spiking signals produced are then conveyed to a motor controller, commanding the robotic arm to escape from a hot source (Fig. 19h). For temperatures that do not pose a threat to the nociceptor, the system does not generate any output signals, and the robotic arm remains stationary. At higher temperatures, the stimulus can be considered a warning, as prolonged exposure may lead to injury. In such cases, spikes are generated with a delay of a few seconds, and the robotic arm is directed to flee upon receiving the spiking signals. For extremely high temperatures, fast spike generation prompts the robotic arm to escape rapidly. Spiking nociceptors contribute to the development of intelligent sensory systems and facilitate the construction of humanoid robots, neural prostheses, and neural interfaces. Despite the advancements, there are challenges in deploying ASNs for nociceptive emulation. Biological nociceptors are proficient at detecting a wide range of painful stimuli and distinguishing between them, showing high sensitivity and selectivity. ASNs-based nociceptors must acquire high level of sensitivity and selectivity to accurately detect and interpret various pain signals. Moreover, nociceptive signals are typically integrated with other sensations to generate an accurate perception of pain, highlighting the importance of integrating ASN-based nociceptive systems with other sensory systems to provide a holistic pain experience. The threshold is pivotal, because it determines the onset of pain perception and subsequent responses. However, this threshold is not fixed and can vary under different conditions. Consequently, developing an ASN-based nociceptor with an adaptive threshold could significantly improve its adaptability to diverse environments. Another critical aspect is biocompatibility when integrating these ASNs into neural interfaces. Ensuring the biocompatibility is crucial to prevent immune reactions, inflammation, or rejection by the body, a factor that has yet to be thoroughly investigated. Lastly, current ASN-based nociceptive perception systems can typically only respond to a single stimulus, which is relatively simple. To emulate the human nociception more accurate, a system capable of responding to multiple stimuli is highly desired.

### Collision Avoidance

**Fig. 20:**
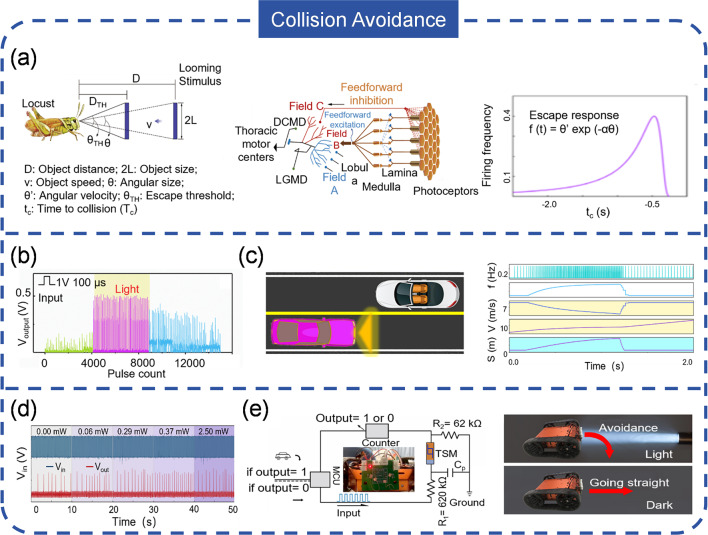
Collision avoidance with ASNs. **a** Working mechanism of collision avoidance of locusts. Reproduced with permission from Ref. [284]. Copyright 2020, Springer Nature. **b** Spiking response of AVN. **c** AVN for meeting control system. **b**–**c** Reproduced with permission from Ref. [289]. Copyright 2021, American Chemical Society. **d** Spiking generation under different light intensities. **e** AVN for real-time collision detection. **d**–**e** Reproduced with permission from Ref. [259]. Copyright 2021, Springer Nature

Collision avoidance comprises a suite of strategies and mechanisms used by organisms to avoid physical contact with obstacles or objects in their environment [282, 283]. This involves sensory systems to detect potential collisions, neural processing to integrate and interpret sensory data, and motor responses to navigate around obstacles. Visual information is particularly significant, with humans relying on binocular vision to estimate distances and insects like locusts using movement detector neurons to detect approaching objects (Fig. 20a). The behavior of movement detector neurons is largely governed by angular velocity and the size of looming objects [284–286]. More specifical, the lobula giant movement detector receives, onto a large dendritic fan, excitatory retinotopic inputs that convey, to a first approximation, the angular velocity of the approaching object. In addition, two dendritic fields arborize in distinct regions of the lobula and descending contralateral movement detector receives phasic nonretinotopic, feedforward inhibition related to object size. Such collective excitatory and inhibitory input leads to a neuronal firing rate that increases, peaks, and decreases when a collision becomes imminent. Conventional vision chips with very-large-scale-integration (VLSI) systems, such as silicon retinas and field-programmable gate array (FPGA) circuits, can implement collision avoidance but face challenges in terms of power and area efficiency [287, 288]. An optional approach is to use AVNs that emulate the firing dynamics of movement detector neurons to perform collision avoidance. Recently, Pei and colleagues reported an AVN to facilitate the self-regulation of speed during the meeting process in driverless vehicles [289]. When exposed to light, it exhibits an increased spiking frequency, indicating its high sensitivity to light (Fig. 20b). Additionally, it is responsive to a range of visible light wavelengths. These characteristics endorse the AVN to be utilized for collision avoidance in driverless cars at night, when the distance between vehicles can be evaluated based on the received light intensity (Fig. 20c). During the meeting process, two driverless cars approach each other, the AVN perceives a higher light intensity when the distance between them decreases. This higher intensity triggers an increase in the ASN’s output frequency. Noted that the car’s speed is controlled by the difference between the maximum output frequency and the AVN’s real-time output during the meeting. Consequently, the vehicle reduces its speed safely. Once the meeting process ends, the light intensity perceived by the ASN decreases and the output spike frequency returns to its initial state, inducing the driverless car resumes its original speed.

Correspondingly, Wang et al. [259] fabricated a biomimetic compound eye with 20 × 20 AVN array, composed of Ag/few-layer black phosphorous nanosheets (NSs)-CsPbBr_3_ perovskite quantum dots (QDs) heterostructure (FLBP–CsPbBr_3_)/indium tin oxide (ITO). The spiking frequency of AVNs can be finely adjusted by the light intensity, with an initial increase followed by a decrease, mimicking the spiking dynamics of movement detector neurons (Fig. 20d). The light response of the AVNs is attributed to the significant enhancement of the surface potential in the FLBP–CsPbBr_3_ layer, as illumination on the CsPbBr_3_ QDs film generates photocarriers. The photogenerated electrons can be readily transferred from CsPbBr_3_ to FLBP through an internal electric field, leaving photo-induced holes in the valence band of the CsPbBr_3_. To enable collision avoidance, a robotic car platform was developed by integrating the AVNs with peripheral circuits (Fig. 20e). A threshold value for the spiking frequency is set to control the robot’s movement: the robot car will deflect to avoid imminent collision when the generated spiking frequency is larger than the threshold value. Although some groundbreaking developments have been reported, the application of ASNs for collision avoidance is still in its early stage. Creating a more biomimetic system demands a profound understanding of neural circuitry and behavior and development of electronic devices. Response time is a critical factor for collision avoidance. Current neuromorphic systems operate at a time scale of seconds, which is much slower than the millisecond response time of biological systems. Moreover, relying solely on visual information to implement the collision avoidance is quite difficult because of the ever-changing environment. Incorporating other types of sensors such as distance detectors with visual sensors can provide a comprehensive and reliable collision detection system.

### Artificial Neural Interfaces

**Fig. 21:**
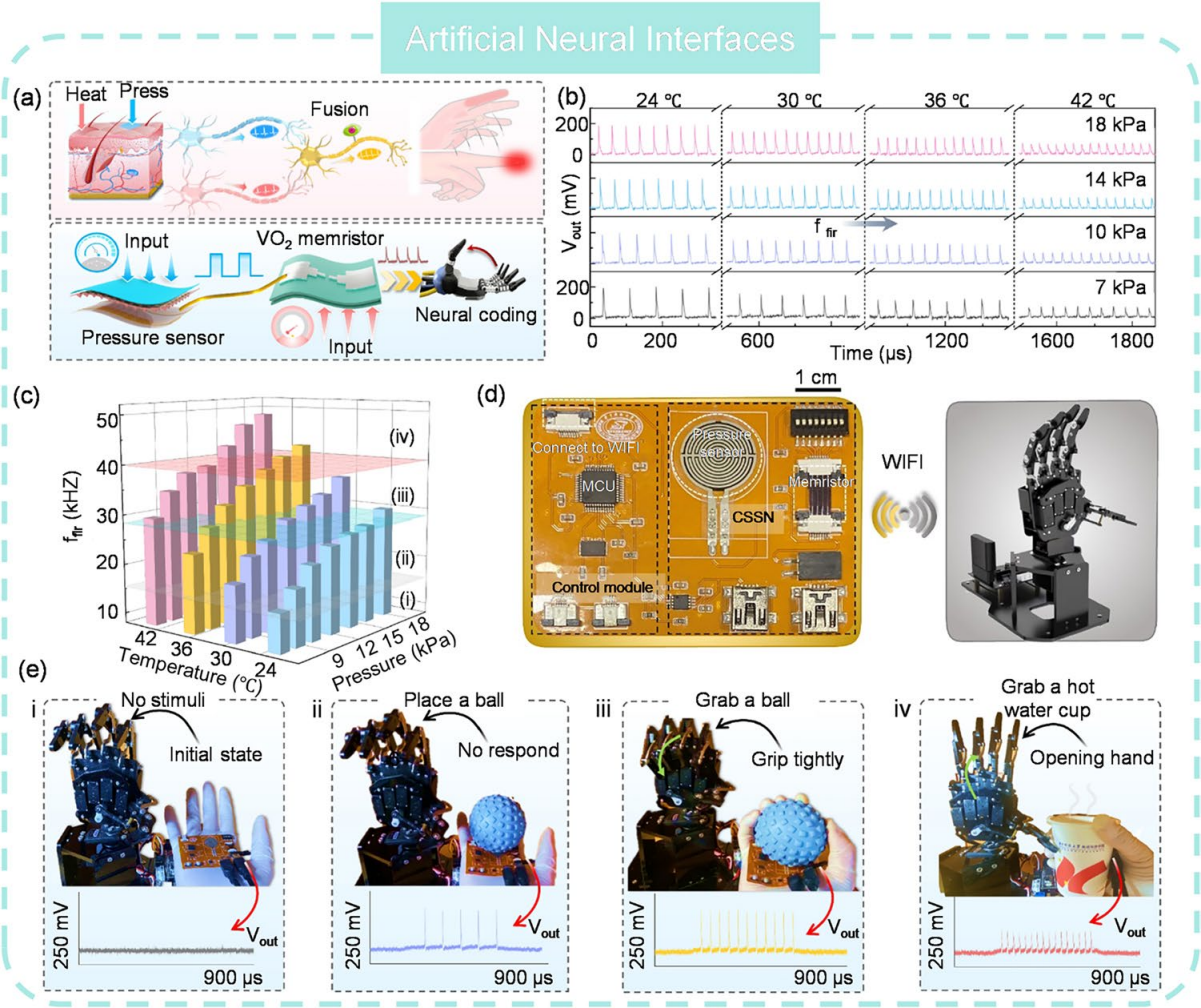
Artificial neural interfaces with ASNs. **a** Biological and artificial neural reflex systems. **b** Spike response of ASN. **c** Systematic investigation of spiking performance of ASN under various conditions. **d** Platform of flexible in-sensor encoding and haptic-feedback system. **e** Real-time feedback of robotic arm under different stimuli. **a**–**e** Reproduced with permission from Ref. [290]. Copyright 2020, Springer Nature

Artificial neural interfaces are systems that establish a connection between the biological nervous system and electrical devices or borrow biological ideas to replicate the neural pathway. A biological neural pathway includes many neurons and synapses to translate the electrical and chemical signals, enabling functions such as sensory perception, processing, and motor responses. Thus, artificial neural interfaces offer solutions to restore lost sensory or motor functions, such as enabling amputees to control prosthetic limbs or helping patients with paralysis to interact with computers through brain-computer interfaces. As discussed above, ASNs can serve as an ideal component to realize artificial neural interfaces. Recently, Li et al. [290] developed a flexible crossmodal ASN based on high-performance VO_2_ memristors and successfully mimic the behavior of the human neural reflex system for human–machine interfaces (Fig. 21a). The ASN can concurrently encode the external thermal and pressure stimulus with spikes (Fig. 21b). The output spiking frequency is systematically studied, and a proportional relationship is established between the spiking frequency and applied stimulus (Fig. 21c). With this ASN, a flexible in-sensor encoding and the haptic-feedback system was presented (Fig. 21d). Under various conditions, the robotic arm can be precisely controlled: when a weak pressure is exerted, the robotic arm keeps still, while if a strong pressure or a high temperature is detected by the ASN, the robotic arm will grasp or loose the object (Fig. 21e). Such flexible spiking sensory-feedback hardware system effectively mimics human gripping and avoidance actions, demonstrating its promising application in human–machine interaction. However, the current artificial neural interfaces are superficial and not a patch on the biological counterpart due to the limited understanding of the biological nervous systems. We can mimic the functionality of biological nervous systems but lack the capability of emulating their complexity of working mechanisms. Therefore, by collaborating with neuroscientists to replicate the dynamics of biological neurons at the molecular level and using a bottom-up strategy to duplicate the electrical and chemical signals transmission, we can finally accomplish artificial neural interfaces that are similar to the real neural pathway.

## Conclusion and Perspectives

**Fig. 22:**
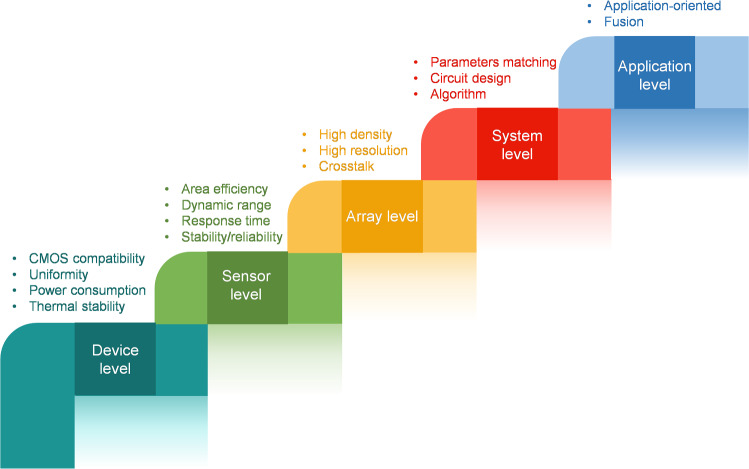
Development roadmap of ASNs from device level to application level

In this review, recent progress of ASNs, which pose key advantages over conventional ADC in respect of hardware cost and power consumption, is introduced and summarized. By emulating biological sensory neurons, ASNs can efficiently detect and convert external information into spikes, showcasing significant potential in artificial intelligence, intelligent sensing, artificial prosthetics, and humanoid robotics. To date, numerous materials, devices, ASNs, and configurations have been proposed to realize tactile, visual, thermal, auditory, olfactory, gustatory, ionic, as well as multimodal encoding, and several intriguing applications have been demonstrated. However, to fully leverage the benefits of ASNs, there are a myriad of concerns that need to be carefully examined at various levels (Fig. 22).

Device level: For the emerging neuromorphic devices that are used to construct ASNs, they should possess the following properties: CMOS compatibility for large-scale fabrication, low operation voltage/current for high energy efficiency, excellent uniformity and endurance for precise encoding, and environmental stability for reliable performance. Although these emerging neuromorphic devices offer clear benefits over CMOS-based devices, none of them currently meet all the requirements necessary for commercializing ASNs. Diffusive memristors can operate at ultralow currents, but their significant variations from cycle to cycle and device to device greatly hamper their applications. Potential solutions such as interface engineering and structure design have been suggested to improve the uniformity by confining/localizing the formation and rupture of conducting filament. However, there is still a lot of room for optimization. Additionally, the materials used in diffusive memristors are not compatible with semiconductor manufacturing processes. It is challenging to scale down and integrate them with other mature technologies. In contrast, Mott memristors demonstrate excellent uniformity through device optimization such as inserting a buffer layer between the top electrode and dielectric layer or carefully tuning the stoichiometry of the Mott insulator. Small arrays of ASNs based on Mott memristors have also been displayed, but large-scale demonstrations are lacking due to their incompatibility with CMOS processes. Furthermore, the operating current of Mott memristors is relatively high (~ mA), resulting in higher power consumption. Changing the electrode or inserting a barrier layer between the oxide and electrode can effectively reduce the current to microamperes, yet it remains uncompetitive with its rivals [159]. Exploring new approaches to lower the power consumption of Mott memristors is essential. STLFETs are 100% compatible with current foundry processing, guaranteeing excellent uniformity and mass production. The device size of STLFETs is larger than memristors, which have a feature size of ~ 4 F^2^, but techniques like 3D integration or gate-all-around (GAA) structures can greatly enhance density [291, 292]. This makes STLFETs the most promising candidates for practical applications. However, the driving voltage of STL MOSFETs is higher (~ 4 V), which poses a potential barrier for low-power sensing. Possible solutions to reduce the driving voltage include reducing the gate length or using materials with narrower bandgaps. For instance, substituting Si (1.1 eV) with InGaAs (0.75 eV) can significantly decrease the operation voltage from 4.2 to 1.3 V [213]. 2D memtransistors shows advantages in visual sensor because of the unique optical properties of 2D materials. Despite that, the large-area growth of 2D materials with high uniformity and quality is tough, which greatly restricts the fabrication of 2D memtransistors at the chip level. Although advanced technologies, for example, chemical vapor deposition (CVD), molecular beam epitaxy (MBE), have been employed to address this issue, a technique that can cover the benefits of cost, materials quality and uniformity is still lacking. Further improve current fabrication technologies and develop novel synthetic techniques is highly required [145]. The ultimate goal of 2D memtransistors is to achieve a complete 2D CMOS chip, which needs to integrate the memtransistors with other peripheral circuits. Thus, there is still a long way due to processing, fabrication, and design constraints. Another common concern for memristors, STLFETs, and 2D memtransistors is thermal instability. Thermal fluctuations can affect the ion movement in memristors, the electron–hole pair generation rate and thermionic emission in STLFETs, and conductivity in 2D memtransistors. It is reported that element doping or materials design is helpful in mitigating thermal effects in memristors, but the thermal issues in diffusive memristors and 2D memtransistors have not been extensively explored and should be further examined.

ASN level: As noted above, ASNs typically include emerging neuromorphic devices and specialized sensors to perform both detection and conversion of external stimuli. When calculating the overall area of an ASN, the area of the sensor itself must be considered. In practice, the sensor area (approximately millimeters squared) is significantly larger than that of the emerging devices (approximately nanometers squared), which can be inefficient and costly. One potential solution to achieve higher compactness in ASN design is to develop a 2-in-1 device that integrates sensing and encoding function. This approach has been successfully implemented for visual encoding using STLFETs, but it has not been widely explored for other sensory modalities. Another possible way is to use advanced microfabrication processing. This allows the fabrication of sensors on a microscale or even nanoscale. In addition, current works are primarily focused on the ability to sense and generate spikes while ignoring the sensing performances. For instance, the dynamic range of ASNs is generally narrower than that of human perception. ATTNs struggle to detect subtle pressures, while the AVNs are difficult to finish the task of high and low light intensity detection. AANs have a limited range of 60–100 decibels, whereas humans can detect sounds as low as 0 decibels. AONs and AGNs is applicable for several types of stimuli, which is far behind its biological counterpart. This challenge can be possibly tackled by carefully matching the performance of sensors and artificial neuronal devices, making the artificial neuronal devices fully respond to the whole dynamic range of sensors. Moreover, an efficient encoding process requires ASNs to operate with fast response times. The response time is largely determined by the sensor itself, as the working speed of the emerging devices is on the nanosecond scale, while the response delay of sensors can be on the millisecond or even second scale. It has been proved that the response time of the sensor can be enhanced by materials design. The stability and repeatability of ASNs are crucial for reliable long-term operation, but these aspects have not been extensively studied in previous works. Given the mature technology and development of various sensors, the stability and repeatability of ASNs are highly dependent on the emerging neuromorphic devices, which may exhibit high endurance but suffer from thermal instability. Enhancing the thermal stability of emerging devices can significantly improve the stability of ASNs. What’s more, there are many reports on ATTNs and AVNs, however, the development of other types of ASNs is insufficient.

Array level: To validate the feasibility of ASNs for real-world applications, realizing their functionality at the array level is imperative. Small-scale arrays have been shown for tactile and visual encoding, but the sensor area remains as large as a square millimeter, which is not cost-effective [293]. The goal to achieve high-density and high-resolution ASN arrays can be realized if several issues are appropriately solved. Firstly, crosstalk between neighboring sensors can lead to unwanted signal propagation and errors in encoding. It can be mitigated by complex readout circuits, but may induce reduced reading speed and increased power consumption. Secondly, the use of massive resistors and capacitors in ASN arrays leads to significant thermal effect because of the IR drop, which degrades performance and causes encoding disturbances. A proper circuit design to avoid excessive heat dissipation is an alternative way to address this issue. Furthermore, multimodal functionality at the array level is yet to be shown. Two potential methods to achieve multimodal sensing are the integration of multiple sensors into a single cell or the detection of multiple stimuli using a single sensor. The former method requires complex structure design, fabrication, and signal readout, which may hinder high-density array integration. The latter approach allows for high-density integration but poses challenges in decoupling multiple stimuli without crosstalk. Given the potential applications in neural prosthetics, artificial afferent/efferent nerves, and wearable systems, the functionality of ASN array on flexible substrates should be investigated, which is rare at present.

System level: ASNs are capable of sensing and encoding information, but they cannot perform data processing, which is crucial for computing and decision-making. To complete the data collection, processing, and feedback loop, ASNs should be coupled systematically with neural networks and actuators. The encoded spikes produced by ASNs serve as input to the neural network, demanding a buffer circuit due to the inability to directly interface with the synaptic device. More importantly, the electrical properties of the synaptic device must align with the output of the ASNs, as the output voltage amplitudes of different devices vary significantly. For instance, diffusive memristor-based sensors have an output amplitude below 1 V, while Mott memristor-based and STL FET-based sensors operate at 1 ~ 3 V and above 4 V, respectively. In some cases, an external amplifier or attenuator circuit may be required to match the voltage levels. It should be pointed out that the interfacing circuits can add to the hardware footprint and power consumption, so they must be carefully designed. Considering ASNs collaborate with spike-based neural network to perform spatiotemporal computations, the development of new efficient algorithms for training the spike-based neural network remains an active research field because the well-established back-propagation algorithm used for deep neural networks is not applicable to spike-based neural networks. To maximize the utility of ASNs in a system context, long-term support from software tailored for neuromorphic computing is necessary. However, unlike traditional computing systems, which are built upon a well-defined hierarchy built on the concept of Turing completeness and the von Neumann architecture, there is no universally accepted system hierarchy or concept of completeness for neuromorphic computing [294].

Application level: ASNs provide an energy-efficient way for signal sensing and converting, which can be used in various applications where sensors are indispensable. A typical scenario is intelligent autonomous systems [295]. Numerous autonomous applications impose stringent limitations on the physical implementation of AI and machine learning for autonomous operations, including restrictions on energy or power consumption as well as requirements for real-time processing. Neuromorphic computing system with ASNs offers one path for resource-constrained intelligent autonomous systems. One challenge for neuromorphic autonomous systems is the limited availability of neuromorphic hardware. Though neuromorphic chips such as Intel’s Loihi, IBM’s TrueNorth, Tsinghua’s Tianjic have been developed, the hardware that can convert the external signals to spikes are quite limited, especially in tactile, olfactory, auditory, thermal and gustatory domain. With the ASNs, this issue can be well addressed and extend the applications of neuromorphic autonomous systems. In order to achieve the desired goal, several requirements of ASNs should be taken into account. First, the ASNs should support the resilient operation of autonomous systems considering the complexity of the work environment. Second, the response of ASNs should be fast to make sure the autonomous systems can process the signals in real time. Third, the ASNs should be capable of implementing the multimodal fusion, which is beneficial for the accurate interaction between the autonomous systems and environments. Forth, it is better for the ASNs to work with traditional sensors to harness their mutual advantages. Here, we illustrate the application of ASNs for autonomous systems. In reality, the requirements of ASNs varies with different applications.

Despite being in its infancy, studies on ASN have showcased immense promising for high energy-efficient sensing and encoding. Collaborations between neuroscientists, device engineers, algorithm developers, software designers, and systems engineers can effectively address the challenges faced by ASNs. It is believed that these joint efforts not only have the potential to significantly advance the application of ASNs but also can propel the development of neuromorphic hardware and systems.
